# Age-stratified health impacts of climate change on older adults in the Western Pacific Region: a scoping review

**DOI:** 10.1016/j.lanwpc.2026.102004

**Published:** 2026-10-23

**Authors:** Dongkyu Lee, In-Hwan Oh, John S. Ji, Mikiko Kanda, Hitomi Kimura, Siwon Lee, Josselyn Mothe, Jinah Park, Enkhtsetseg Shinee, Ho Kim

**Affiliations:** a Institute of Health and Environment, Seoul National University, Seoul, Republic of Korea; b Department of Preventive Medicine, University of Ulsan College of Medicine, Seoul, Republic of Korea; c Vanke School of Public Health, Tsinghua University, Beijing, China; d Healthy Ageing Unit, Division of Data, Strategy and Innovation, World Health Organization (WHO) Regional Office for the Western Pacific, Manila, Philippines; e WHO Asia-Pacific Centre for Environment and Health in the Western Pacific Region, Seoul, Republic of Korea; f Department of Public Health Sciences, Graduate School of Public Health, Seoul National University, Seoul, Republic of Korea

**Keywords:** Climate change, Western Pacific, Older adults, Young-old, Old–old, Mortality

## Abstract

While research on the health impacts of climate change is growing, studies integrating these effects across finer age strata within older adult populations, such as distinguishing young-old from old–old adults remain scarce, particularly in the Western Pacific region. This scoping review synthesizes 119 cases extracted from 100 peer-reviewed studies published from 2016 to 2025, identified via systematic searches of PubMed, Scopus, and Embase using keyword clusters for older adults, climate change, and the Western Pacific. Most cases (n = 61, 51%) examined heat, followed by extreme cold (n = 31, 26%), air pollution (n = 11, 9%), with geographic concentration in mainland China (n = 84, 71%); old–old adults generally showed greater vulnerability than young-old adults. These findings suggest heterogeneity in climate impacts among older adults, indicating that a one-size-fits-all approach may be insufficient and that finer age stratifications could help inform policies better tailored to old–old adults.

**Research in context**

**Evidence before this study**

Alongside growing concerns about climate change, vulnerability among older populations has been widely discussed. However, rapid population aging in the Western Pacific Region (WPR) has highlighted the need to move beyond defining older adults simply as those aged 65 years and above, and to consider heterogeneity of vulnerability within older populations. Nevertheless, studies that stratify older age groups to examine age-specific vulnerability in the context of climate change remain limited.

**Added value of this study**

This scoping review provides the first comprehensive synthesis of how climate change affects health outcomes across different age groups among older populations within the WPR. Based on an analysis of 119 cases extracted from 100 peer-reviewed studies, the findings indicate that most studies examined heat with geographic concentration in mainland China. In addition, the health impacts of climate change are unevenly distributed among older adults. Substantial heterogeneity exists within this population, with old–old adults generally demonstrating a higher point estimate of vulnerability with this difference reaching statistical significance in many, though not all, comparisons.

**Implications of all the available evidence**

The findings emphasize that older adults are not a homogeneous group, as those in advanced age are generally more vulnerable to the health impacts of climate change. Given the age-specific variations in health risks and the region's rapid population aging, further research is needed to clarify these heterogeneous effects, particularly across geographic contexts beyond mainland China and climate change threat beyond heat. Policymakers and practitioners in the Western Pacific Region could benefit from recognizing varying vulnerability across older adults' subgroups and develop tailored adaptation strategies that focus on old–old adults.

## Introduction

Climate change is generally recognized as a serious threat to global health, especially among older adults in the Western Pacific region (WPR).^1^, ^2^, ^3^, ^4^, ^5^, ^6^ Older adults are particularly vulnerable to climate change-related events because they face heightened health risks from ageing-related physiological changes, such as reduced sweating and fluid dysregulation, chronic diseases, polypharmacy impairing thermoregulation, cognitive impairment, and social isolation.^7^, ^8^ These amplify vulnerability to extreme heat and cold, natural disasters, and air pollution, exacerbating heat stroke or cold spell, cardiovascular events, and neurodegenerative diseases. Sociodemographic inequities and ageism further increase exposure and hinder recovery.^7^ The WPR, encompassing both large nations and small island countries, faces climate-related challenges that adversely affect public health. Numerous studies have documented associations between climate change-related extreme weather events and health outcomes in Australia and China,^4^, ^5^ and while evidence remains limited, Pacific island countries are similarly affected.^6^

The population in the WPR is rapidly aging, with the number of people aged 60 years or over projected to double from 2020 to 2050.^9^ This demographic shift is occurring alongside intensifying climate change impacts, including more frequent and severe extreme weather events such as heatwaves, tropical cyclones, storms and droughts.^10^ Understanding how climate change impacts on the disease burden among older adults is crucial because it highlights the intersection of two major global megatrends, population aging and climate change, both priorities for the WPR.^11^

Despite the convergence of climate change and population aging in this region, there remains a paucity of synthesized evidence. While age is among the most frequently discussed effect modifiers of vulnerability to climate change, comprehensive reviews that elucidate when and how age-related effects are most pronounced, particularly among older adults, remain limited.^12^ Although several studies have examined the health impacts of climate change, most have focused on regions beyond, or only partially within, the Western Pacific,^4^, ^6^, ^13^, ^14^ or explored single climate-related threat.^15^ Some studies have specifically targeted older adults,^1^, ^2^, ^4^ yet these have commonly treated this population as a homogeneous group, with limited consideration of how climate change exerts differential health impacts across age subgroups within older populations. For instance, young-old adults may not differ substantially from the general adult population, potentially masking the specific vulnerabilities of old–old adults, who are generally frailer and more physiologically and socially susceptible to the adverse effects of extreme climatic conditions. Addressing these potentially heterogeneous impacts requires a comprehensive understanding of how climate change influences the health and well-being of older adults.

This study aims to synthesize peer-reviewed evidence on the health impacts of climate change among older adults in the WPR. Specifically, this scoping review seeks to identify and characterize heterogeneity in these impacts across different age subgroups within the older population.

## Search strategy and selection criteria

### Study design and framework

This scoping review followed the Joanna Briggs Institute (JBI) methodology for scoping reviews and was guided by the PRISMA-ScR checklist (Table A1).^16^, ^17^ This review was not registered in a public registry.

### Search strategy

Our keyword selection was grounded in the list of “Threats related to climate change and their impacts on the health and well-being of older people” presented in WHO (2022),^18^ so as to comprehensively capture the major climate-related threats. This list identifies extreme and prolonged heat or cold, air pollution, rising sea levels, and extreme weather events (storms, typhoons, and hurricanes), as well as vector-borne and zoonotic diseases. It was further supplemented with wildfire and drought, which have also been recognized as distinct climate hazards in previous studies.^2^, ^19^

A search was conducted across PubMed, Scopus, and Embase combining keyword clusters for climate change, older adults, and the WPR using the Boolean operator AND (Table A1). Unlike other terms, air pollution and vector-borne/zoonotic disease are primarily governed by non-climatic determinants (e.g., emission rates, vector ecology, control policy); therefore, their search terms were required to co-occur with a climate-related term cluster (e.g., “climate change,” “climate variability”) using the AND operator, to ensure relevance and keep the search yield manageable. The search was conducted on 19 June 2026. Given the breadth of the search terms, backward and forward citation searching was not performed.

### Eligibility criteria

Eligible studies quantitatively assessed the association between climate-related threats and health outcomes among older adults within the WPR, reported in comparable terms.

The list of climate-related threats followed the definition of WHO (2022),^18^ and health outcomes were defined as any health status measure, or health and long-term care utilization. Studies covering populations beyond older adults, or regions beyond the WPR, were retained only if results were stratified such that data for the older adult subgroup and the WPR region could be separately extracted. Comparability required a consistent metric across subgroups (e.g., rates or proportions with a defined denominator, rather than raw counts alone); for instance, a study reporting only the number of fatalities by age subgroup, without a population size was excluded, since raw counts alone cannot establish which subgroup was disproportionately affected. Studies were restricted to English-language, peer-reviewed original research or review articles published between 2016 and 2025; gray literature was excluded to ensure the reliability of the estimates being compared.

### Study selection and data extraction

Study selection was conducted in two stages: (1) title and abstract screening and (2) full-text review. Both stages were performed independently by two reviewers, who categorized articles as “include,” “exclude,” or “unsure.” Discrepancies were resolved through discussion, with a third reviewer consulted if consensus could not be reached.

A standardized data extraction template, developed and piloted by the research team, was used to collect information from the included studies. Extracted fields were author(s), year, country, population/setting, climate threat category, age-subgroup comparison structure, outcome measures, and key findings. Data were compiled in Excel and summarized in both tabular and narrative formats, organized by climate threat and health outcome. Investigators were not contacted to obtain or confirm data.

### Quality assessment and evidence tiers

In line with scoping review guidelines, formal quality appraisal and risk-of-bias assessment were not conducted.^16^ Instead, to gauge the certainty of evidence for vulnerability among old–old adults, we classified included studies into three tiers based on the comparison structure underlying their estimates.

Tier 1 studies reported subgroup estimates without a formal statistical comparison. Tier 2 studies formally tested differences against a younger reference group (generally aged <65 years). Tier 3 studies formally tested differences using an older-adult subgroup as the reference, or compared all older-adult subgroups simultaneously; studies tested against both a younger reference group and within older adults were also classified as Tier 3, as this best captures the within-group age gradient. We prioritized formal comparisons where available (Tiers 3 and 2) and, in their absence, relied on the significance of subgroup-specific estimates (Tier 1), using point estimates alone only when confidence intervals were unavailable. Full decision rules are provided in Appendix Table A2.

## Results

### Characteristics of included studies

The study selection process is illustrated in the PRISMA flowchart (Fig. 1). For this scoping review, three databases were searched, PubMed, Scopus, and Embase, yielding 544, 2440, and 491 records, respectively. After removing 864 duplicates, 2611 unique records remained. Following title and abstract screening based on predefined eligibility criteria, 2047 records were excluded, leaving 564 for full-text assessment. Of these, 8 records could not be retrieved, and 469 articles were excluded during full-text review: 443 studies lacked subgroup analysis among older adults, and 26 studies reported older adult subgroup results but in non-comparable forms. This resulted in 87 original study articles. An additional 13 studies, identified before the search strategy was finalized and not captured by the finalized strategy described above, were retained based on the eligibility criteria. Consequently, 100 original articles were included in this review; no relevant review papers were found. Studies included in this review are listed in the Supplementary Material (Appendix Table A3), and are cited herein and denoted by the prefix “a.”

**Fig. 1:**
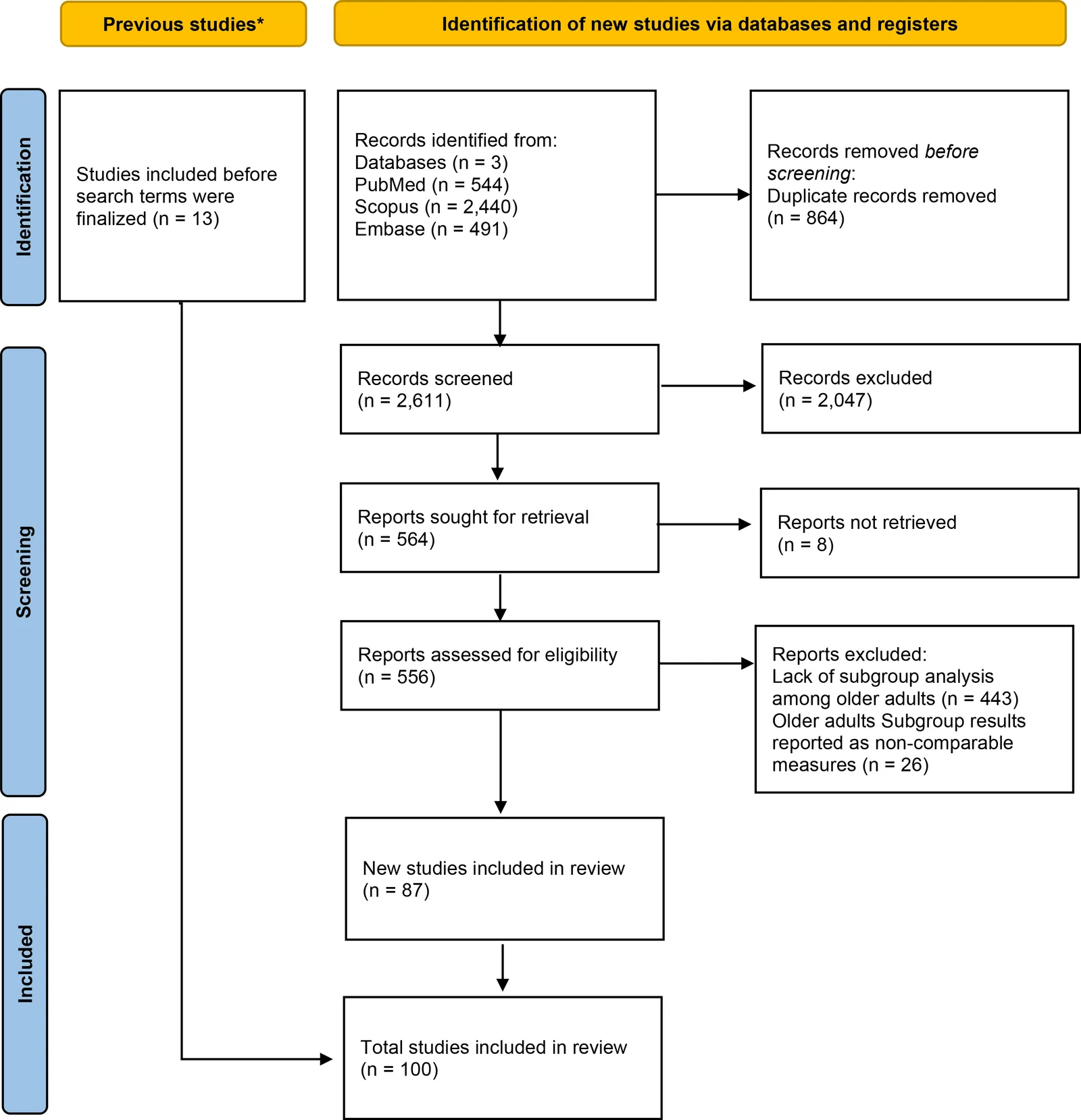
**PRISMA flowchart showing flow diagram of study selection**. ∗Previous studies refer to studies that were identified before the search terms were finalized.

Fig. 2 shows the number of included studies by year of publication. With the exception of a peak in 2023 (25 studies), the number of relevant studies generally increased from 6 in 2016 to 16 in 2025.

**Fig. 2:**
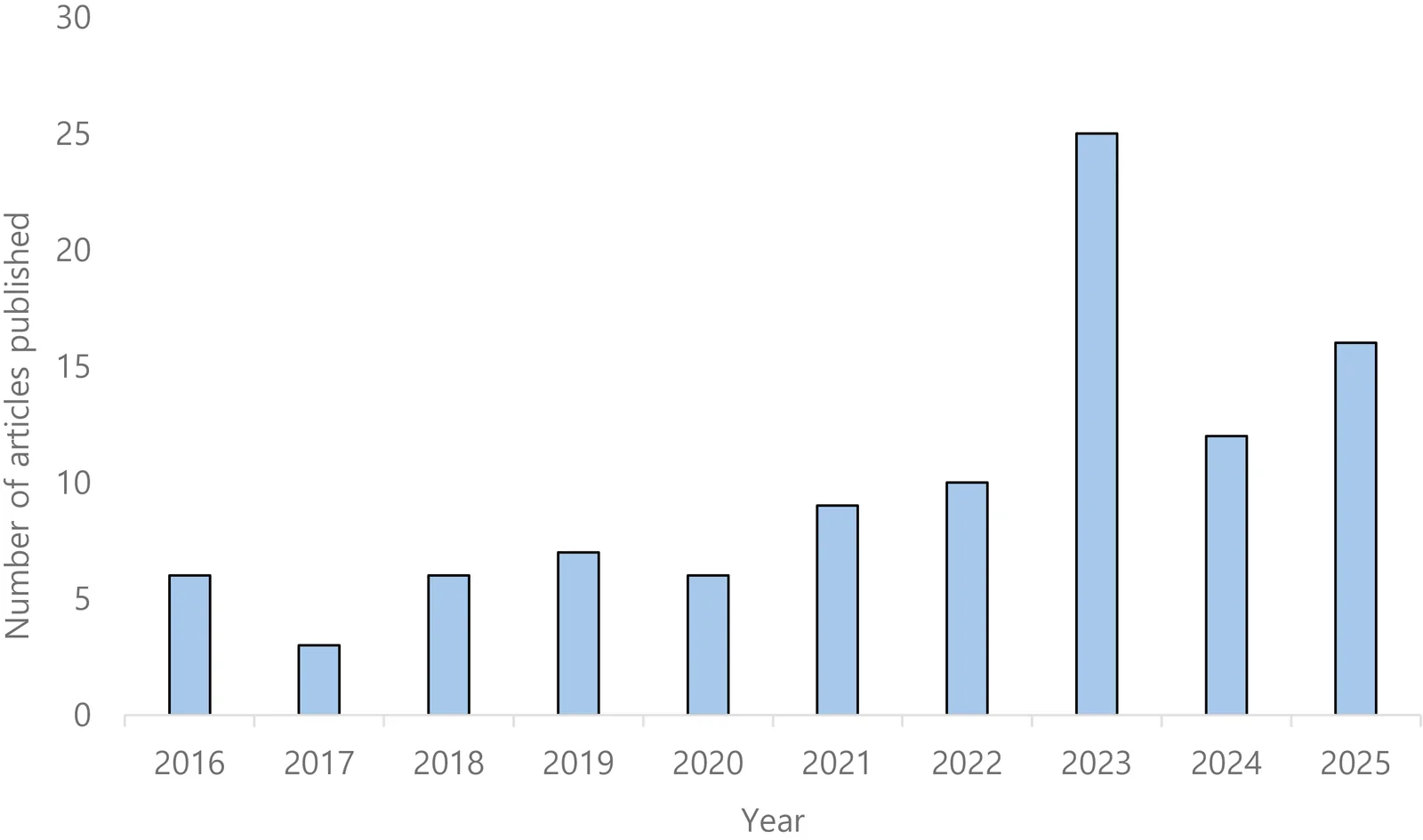
**Number of studies by year of publication, 2016–2025**.

Table 1 presents the countries and regions in which the included studies were conducted. Although all countries and areas in the WPR were considered for the database search, the studies meeting the predefined inclusion criteria originated from only a limited subset of locations: mainland China (70), Japan (10), Australia (8), Hong Kong Special Administrative Region (7), Republic of Korea (2), Malaysia (1), and Vietnam (1), in addition to one multi-country (global) study (1).

**Table 1:** Countries or regions where the studies were conducted.

Country	Studies		Cases	
	n	%	n	%
China	70	70	84	71
Japan	10	10	12	10
Australia	8	8	10	8
China, Hong Kong SAR	7	7	7	6
Republic of Korea	2	2	3	3
Malaysia	1	1	1	1
Vietnam	1	1	1	1
Global	1	1	1	1
TOTAL	100	100	119	100

Table 2 summarizes the 119 cases extracted from the 100 included studies by threat. Extreme heat was most common (n = 61, 51%), followed by extreme cold (31, 26%), air pollution (n = 11, 9%), and temperature variability (n = 9, 8%) and extreme weather (n = 7, 6%). Individual study-level effect estimates are in Appendix Table A3.

**Table 2:** Increasing vulnerability with age among older adults by climate-related threats and health outcomes.

Threats	Health outcomes	Share (n)	Threats	Health outcomes	Share (n)
Extreme heat	Mortality	30/42	Extreme Weather	Mortality	1/3
	Other health status	0/1		Other health status	0/2
	Hospital admission	4/7		Hospital admission	0
	Emergency service use	4/10		Emergency service use	0
	Other service use	1/1		Other service use	0/2
	All health outcomes	39/61		All health outcomes	1/7
Extreme cold	Mortality	17/21	Air pollution	Mortality	1/3
	Other health status	0		Other health status	0/2
	Hospital admission	3/3		Hospital admission	2/4
	Emergency service use	4/6		Emergency service use	1/1
	Other service use	1/1		Other service use	0/1
	All health outcomes	25/31		All health outcomes	4/11
TV	Mortality	1/2	All Exposure Pathways	Mortality	50/71
	Other health status	0/3		Other health status	0/8
	Hospital admission	1/2		Hospital admission	10/16
	Emergency service use	1/2		Emergency service use	10/19
	Other service use	0		Other service use	2/5
	All health outcomes	3/9		All health outcomes	72/119

Note: Analyses are based on 119 cases extracted from 100 studies. Share indicates the number or percentage of cases which shows increasing vulnerability with age among older adults.

### Synthesis of the findings

In this review, climate change threats were categorized into five groups: extreme heat, extreme cold, temperature variability (hereinafter, TV), air pollution, and extreme weather. Health outcomes were classified as mortality, other health status, hospital admission, emergency service use, or other service use.

To illustrate age-related variation in climate change impacts, findings from the 119 cases were grouped and summarized by comparing young-old and old–old adults. Although age boundaries within older adults varied across studies, this review broadly labels the relatively older subgroup as old–old adults (generally aged ≥75 years) and the relatively younger subgroup as young-old adults (generally aged 65–74 years), for descriptive consistency; these labels are relative rather than fixed. When more than two age groups were reported, the oldest was treated as the older group; otherwise, the study's own original strata were used.

Applying the tier-specific decision rules described in the Methods, each study was labeled “Yes” in the Age Gradient column when vulnerability increased with advancing age within the older-adult population, “No” when it did not, and “Mixed” when estimates pointed in opposite directions across threat metrics or population strata (e.g., PM_2.5_ versus PM_10_; men versus women), with individual study-level classifications and the effect estimates on which they were based reported in Table A3. If studies reported multiple outcomes (e.g., across lags), the measure primarily discussed by the authors was recorded as primary, or cumulative values if none was specified. Pooled estimates were used when available across multiple years; otherwise, the most recent results were selected. Where multiple outcomes were equally relevant, both were included.

#### Extreme heat

Extreme heat was the most frequently examined climate-related threat (n = 61) among the cases identified in this review. Most cases focused on high ambient temperatures,a3,a4,a9,a10,a12,a18,a21,a24,a27,a28,a31,a33,a37,a38,a39–a41,a44,a47,a50,a51,a52,a54,a59,a64,a68,a70,a75,a76,a80,a81,a85,a89,a92,a94,a96,a99 followed by heatwaves.a2,a14,a20,a30,a55,a60,a66,a69,a72–a74,a77,a78,a82,a87,a91,a93,a98,a100 A smaller number of cases assessed joint hot events, such as compound hot-dry events (CHDEs) or the combination of heatwaves and high ozone concentrations,a67,a84,a97 while a few examined compound hot extremes, defined as sequential hot nights and hot days occurring in either order.a34,a35 Regarding health outcomes, mortality was the most commonly evaluated,a2,a3,a9,a14,a18,a20,a21,a30,a35,a38,a39,a41,a44,a50,a52,a54,a55,a59,a60,a64,a67,a68,a70,a72,a73,a74,a75,a76,a77,a78,a80,a82,a84,a85,a87,a91,a92,a94,a96,a97,a99,a100 followed by emergency service use,a12,a24,a27,a31,a33,a34,a37,a47,a81,a89 hospital admission,a4,a10,a28,a40,a51,a69,a93 other status use,a66 and other service use,a98 as shown in Table 2.

Among cases that formally tested differences between older-adult subgroups, 5 of 11 showed vulnerability increasing with age (Tier 3). Among cases that formally tested differences against a younger reference group, 5 of 6 showed this pattern (aged <65 years; Tier 2). Both Tiers provide formal statistical support, but Tier 3 offers stronger evidence by directly comparing older-adult subgroups, whereas Tier 2 compares each subgroup only against a younger reference, not against each other. The remaining cases belonged to Tier 1, which relied on the significance of individual subgroup-specific estimates in the absence of a formal statistical comparison across subgroups. Of the 44 Tier 1 cases, 29 showed an age gradient. Overall, 39 of 61 cases (64%) examining extreme heat showed evidence of increasing vulnerability with age among older adults (Table 3). Extreme heat significantly increased mortality among old–old but not young-old adults in some cases.a55 One case found CHDEs significantly increased mortality in both subgroups, but the effect was significantly larger among old–old adults than younger generations (aged <65).a84 Under less severe conditions, where only a heatwave occurred, significantly large impacts were observed only among old–old individuals.a74

**Table 3:** Increasing vulnerability with age among older adults by climate-related threats and comparison tiers.

Threat	Comparison tier	Share (n)	Threat	Comparison tier	Share (n)
Extreme heat	3	5/11	Extreme weather	3	0/5
	2	5/6		2	0
	1	29/44		1	1/2
	All	39/61		All	1/7
Extreme cold	3	3/3	Air pollution	3	0/2
	2	3/3		2	1/2
	1	19/25		1	3/7
	All	25/31		All	4/11
Temperature variability (TV)	3	2/3	All threats combined	3	10/24
	2	1/3		2	10/14
	1	0/3		1	52/81
	All	3/9		All	72/119

Note: Analyses are based on 119 cases extracted from 100 studies. Share indicates the number or percentage of cases which shows increasing vulnerability with age among older adults.

#### Extreme cold

Cold was the second most frequently examined climate change threat (n = 31). Among them, most focused on ambient cold temperatures,a3,a6,a8,a18,a21,a27,a28,a33,a38,a43,a44,a47,a51,a54,a76,a80,a85,a89,a99 followed by cases examining cold spells,a5,a19,a25,a39,a71,a79,a82,a98 and a small number of compound cold casesa65,a95 and hypothermia cases. ^a11, a23^ Regarding outcomes, mortality was the primary measure,a3,a5,a6,a18,a19,a21,a25,a38,a39,a43,a44,a54,a65,a71,a76,a79,a80,a82,a85,a95,a99 followed by emergency service use,a11,a23,a27,a33,a47,a89 hospital admission,a8,a28,a51 and other healthcare service use.a98

Extreme cold showed the most pronounced age-related increase in vulnerability among older adults, compared with the other climate change threats examined. Notably, every study that applied a formal statistical test showed this pattern: all 3 cases that formally tested differences between older-adult subgroups (Tier 3) showed vulnerability increasing with age, as did all 3 cases that formally tested differences against a younger reference group (Tier 2). The remaining cases belonged to Tier 1, which relied on the significance of individual subgroup-specific estimates; of these, 19 of 25 showed an increasing age gradient. Overall, 25 of 31 cases (81%) examining extreme cold showed evidence of increasing vulnerability with age among older adults (Table 3). Several Tier 2 cases illustrate how formal comparisons against a younger reference group revealed increasing vulnerability with age under different cold exposure definitions. In a study of ambient cold exposure, mortality risk increased across age groups (65–74 years: OR 1.04 [95% CI 0.95–1.15], non-significant; 75–84 years: OR 1.19 [1.10–1.30]; ≥85 years: OR 1.33 [1.22–1.45]), with the formal comparison against the <65-year reference group reaching significance for the two older subgroups but not for the youngest.a6 A study examining cold spellsa19 similarly found elevated mortality risk in both the 65–74 and ≥ 75 age groups (RR 1.46 [1.33–1.59] and 1.62 [1.42–1.81], respectively), with a formal test confirming a significant age gradient relative to the 0–64 reference group (P = 0.015). A third study examined compound dry-cold and wet-cold events separately and found that mortality risk increased with age under both exposure types, with the age gradient reaching significance in the oldest subgroups (≥85 years) for both dry-cold (RR 1.16 [1.10–1.22], P = 0.005) and wet-cold events (RR 1.34 [1.27–1.41], P < 0.001), although the formal comparison was not significant for the youngest old-age subgroups under either exposure type.a65

#### Temperature variability

Among nine identified cases, four investigated intra-day variability exclusively through the diurnal temperature' range (DTR),a1,a36,a86,a88 and four assessed intra-day and inter-day temperature variability,a17,a48,a53,a61 and one examined long-term temperature variability.a63 Regarding health outcomes, three examined mortalities,a53,a61,a86 followed by hospital admission,a17,a48 emergency service use,a1,a36 and other health status.a63,a88

In general, the age gradient in vulnerability was less pronounced for temperature variability than for other climate-related threats. Overall, 3 of 9 cases (33%) showed evidence of increasing vulnerability with age, with the proportion being higher among Tier 3 cases, where formal comparisons between older-adult subgroups were made (2 of 3, 67%). One case provided an in-depth examination of the modifying effects of age and sex.a1 It showed that among adults aged 65 years and older, only those aged 75+ exhibited significant associations between DTR and emergency room admissions for cardiovascular diseases, whereas aged 65–74 subgroup did not. In addition, the estimated effects of DTR among females aged 75+ were approximately 1–2 times higher than those among males. Furthermore, the differences between the 65–74 and 75+ age groups within the same sex were significant only among females (P < 0.05), suggesting that old–old adults are more vulnerable than young-old adults to variations in DTR, particularly among females. However, another case found no significant differences across age groups within older adults.a88

#### Air pollution

Among the eleven identified air pollution-related cases, six assessed particulate matter alone (PM_2.5_ and PM_10_),a7,a16,a22,a29,a32,a46 three assessed both PM and other pollutants,a13,a15,a26 and two focused on ozone.a45,a90 Among the health outcomes examined, four cases examined hospital admission,a7,a15,a16,a32 three examined mortality,a13,a26,a46 and two examined other health status measures such as cognitive impairment and cardiovascular disease prevalence,a45,a90 followed by emergency service usea29 and other healthcare service use.a22

In general, the age gradient in vulnerability for air pollution was intermediate, less pronounced than for extreme heat or cold but more pronounced than for the other climate-related threats. Overall, 4 of the 11 cases (36%) showed evidence of increasing vulnerability with age, while among the two cases that compared subgroups within older adults (Tier 3), no significant differences were observed.a22,a45

In air pollution, findings were mixed across cases, with results sometimes diverging on whether vulnerability increased with age. One possible explanation is the wide variation in how pollutants are defined and measured. For instance, one case found that when particulate matter was measured as PM_2.5_, the risk of hospitalization was not significant in either young-old or old–old adults. When measured as PM_10_, however, the point estimate was significant for old–old adults but not for young-old adults (Tier 1); the formal comparison against the younger reference group similarly indicated significantly greater risk among old–old adults (Tier 2).

#### Extreme weather

This section addresses climate-related threats beyond extreme heat, extreme cold, and air pollution, including extreme weather events (storms, typhoons, and hurricanes), vector-borne or zoonotic diseases, wildfire, and drought. The search identified seven eligible cases: four addressed floods,a42,a49,a56,a58 and one each addressed typhoon,a83 wildfire,a57 and general climate change exposure.a62 No cases examining vector-borne or zoonotic diseases were identified. While no cases examining drought in isolation were identified, several cases assessed the compound effects of dryness combined with extreme heata67,a84 or extreme cold.a65 In these cases, vulnerability increased with age and were accordingly covered in the respective heat or cold sections. Among the health outcomes, other health statusa57,a58,a62 was most frequently assessed, followed by mortalitya49,a83 and other healthcare service use.a42,a56

In general, the age gradient in vulnerability was least pronounced in the extreme weather section: of the seven included cases, only one flood case (Tier 1) showed increasing vulnerability with age,a49 while the remaining cases generally showed no such relationship (1/7, 14%). For example, one case examined the effects of climate change-related events (bushfires, floods, or heatwaves) on mental health,a62 finding that climate impacts were significantly higher among young-old adults compared with old–old adults. Another case assessed the impact of flooding on nursing home admissions,a56 reporting relatively higher estimated rates among young-old adults. Both cases thus found young-old adults to be more affected than old–old adults by climate-related events.

#### Temporal trend

A small number of cases reported temporal trends in the health impacts of extreme heat and cold among older adults (Table 4).a28,a70,a82 These cases generally show declining vulnerability to extreme cold across both young-old and old–old adult subgroups, although this pattern was not consistent across all age groups and outcomes. Findings for extreme heat were mixed, with some cases reporting increasing risk and others reporting no significant change or a decline. Given the small number of cases included, these patterns should be interpreted as suggestive.

**Table 4:** Temporal changes in significance of age-group vulnerability to heat/cold events.

Threat/Outcome	Measure	Age groups	Period	Effect estimates (point est, 95% CI)	Difference between period	Ref
Heat/Hospital admission	RR	60–69	1995–2016	1.01 (1.00, 1.03), NS	<0.001	a28
			1995	0.91 (0.88, 0.94), sig		
			2016	1.09 (1.06, 1.12), sig		
		70–79	1995–2016	1.01 (1.00, 1.02), NS	<0.001	
			1995	0.95 (0.92, 0.97), sig		
			2016	1.07 (1.04, 1.10), sig		
		80+	1995–2016	1.00 (0.98, 1.01), NS	<0.001	
			1995	0.95 (0.92, 0.99), sig	(P for trend)	
			2016	1.03 (1.00, 1.05), NS		
Heat/Mortality	ASR	50–69	1990	1.15 (no CI)	−1.23 (−2.03, −0.42)	a70
			2019	0.76 (no CI)		
		70+	1990	9.02 (no CI)	0.37 (−0.49, 1.22)	
			2019	9.09 (no CI)	(EAPC in ASR)	
Heat/Mortality	RR	65–74	2006–2019	1.05 (0.87, 1.27), NS		a82
		–	2006	1.09 (0.81, 1.45), NS	0.660	
		–	2019	0.98 (0.74, 1.29), NS		
		75+	2006–2019	1.21 (1.10, 1.33), sig		
		–	2006	1.19 (1.02, 1.39), sig	0.700	
		–	2019	1.14 (1.00, 1.31), sig	(P for trend)	
Cold/Hospital admission	RR	60–69	1995–2016	0.98 (0.97, 1.00), NS	0.046	a28
			1995	1.01 (0.98, 1.04), NS		
			2016	0.97 (0.94, 0.99), sig		
		70–79	1995–2016	1.03 (1.01, 1.04), sig	<0.001	
			1995	1.11 (1.08, 1.14), sig		
			2016	0.96 (0.94, 0.99), sig		
		80+	1995–2016	1.07 (1.06, 1.09), sig	0.499	
			1995	1.08 (1.05, 1.12), sig	(P for trend)	
			2016	1.07 (1.04, 1.09), sig		
Cold/Mortality	RR	65–74	2006–2019	1.15 (1.02, 1.30), sig		a82
		–	2006	1.27 (1.09, 1.47), sig	0.040	
		–	2019	1.01 (0.86, 1.19), NS		
		75+	2006–2019	1.27 (1.13, 1.42), sig		
		–	2006	1.37 (1.19, 1.57), sig	<0.001	
		–	2019	1.20 (1.10, 1.31), sig	(P for trend)	

Note: RR, relative risk; ASR, age-standardized rate; EAPC, estimated annual percentage change.

## Discussion and conclusion

### Summary of the results

This review aimed to map existing research on the health impacts of climate change among older adults in the WPR, with a particular focus on variations in impacts within this population, synthesizing evidence from 119 cases extracted from 100 peer-reviewed studies published between 2016 and 2025. It uniquely highlights heterogeneity in vulnerability, distinguishing old–old from young-old adults, which could inform targeted policy and research.

This review reveals significant gaps in climate-related threats and geographical coverage; research on this topic has been concentrated in a limited number of climate change threats and countries within the WPR. Most cases (n = 61, 51%) examined extreme heat, with geographic concentration in mainland China (n = 84, 71%). While our search strategy followed the WHO framework, which covers a broad range of climate-related threats,^18^ the included studies were concentrated on heat, cold, and extreme weather events; no studies were identified examining age-stratified impacts of rising sea levels or vector-borne disease among older adults. Furthermore, although the WPR encompasses diverse settings, only a limited number of countries and areas were represented in this review, and no studies from Pacific island nations were identified. These gaps are unlikely to be coincidental, as sea-level rise and vector-borne disease risk are generally more pronounced in Pacific island nations. The absence of such studies likely reflects limitations in data availability for age-stratified climate-health research among older adults in the region.

Older adults are generally recognized as more vulnerable to climate-related events than younger populations. The studies included in this review further indicate that, among older adults, old–old adults are generally at greater risk than young-old adults, although this pattern was not always statistically significant. This review also reveals that the presence of an age gradient varies substantially across climate-related threats: it was most pronounced for extreme cold and least pronounced for extreme weather events. Among within-older-adult comparisons (Tier 3), all 3 extreme cold cases showed increasing vulnerability with age, whereas none of the 5 extreme weather cases confirmed such a gradient among older adults. A similar pattern emerged among the 14 cases that formally tested differences against a younger reference population (Tier 2): 10 showed vulnerability increasing with age, and of these, 9 showed a significant difference from the younger reference group only among adults aged 75 years and older, not among those aged 65–74 years. This pattern is consistent with prior proposals to define advanced old age from 75 years onward,^20^, ^21^ suggesting that the age at which climate-related vulnerability becomes statistically distinguishable from younger populations may itself begin closer to 75 years (Table A3).

A non-significant age-related increase does not imply a uniform decline in vulnerability with age; the relationship may instead be non-monotonic, or a true difference may simply lack the power to reach significance, although some included studies did show vulnerability decreasing with age. The outcome measure may also shape the observed pattern: where absolute risk among older adults is already high, relative risk measures may show a smaller apparent age-related increase even when the absolute increase is substantial.^22^, ^23^ Explanations for this reverse pattern remain study-specific, with no systematic account in individual studies, prior reviews of climate change and health^4^, ^5^, ^6^, ^13^, ^14^, ^24^ or reviews of specific threats such as heat,^8^, ^15^ warranting further research.

A further notable finding regarding temporal trends is that older adults appear to have become less vulnerable to extreme cold, particularly among young-old adults, whereas heat trends were mixed, with some cases showing increasing risk and others no change or decline. Declining cold vulnerability may reflect improved adaptation policies, while the mixed heat pattern may indicate uneven adaptation to increasingly intense heat events. These findings are broadly consistent with previous studies, which project increasing heat-related and declining cold-related mortality among older adults.^25^

Several mechanisms likely underlie higher vulnerability among old–old adults compared with young-old adults. Age-progressive physiological declines, including impaired thermoregulation, higher burden of chronic conditions and polypharmacy, and greater functional disability, reduce old–old adults’ capacity to respond to and recover from climate-related health threats.^7^, ^8^ In the WPR, these vulnerabilities are further compounded by social factors such as low income, rural residence, and social isolation, which limit adaptive capacity.

Despite this evidence, most current policy frameworks still conceptualize older adults as a single, undifferentiated group, typically defined as those aged 60 or 65 years and over. WHO regional documents and the Regional Action Plan on Healthy Aging in the Western Pacific, for example, highlight the rapid growth of the population aged 65+ and the need to make health and care systems more responsive to older people, but rarely distinguish among subgroups within older adults.^26^ Similarly, global and regional reports on climate change and aging in Asia–Pacific tend to describe “older persons” or “people aged 60+” as a broad vulnerable category, without systematic attention to within-group heterogeneity by age, functional status or living arrangement.^27^

This review's finding that vulnerability differs systematically between young-old and old–old adults suggests that such homogenization may leave the old–old inadequately addressed by current frameworks. This concern is amplified by the scale of population aging in the WPR: WHO estimates indicate that more than 240 million people aged 65 years and older currently live in the region, a number expected to double by 2050, while the population aged 75 years and above is projected to more than triple over the same period.^9^ At the same time, the Lancet Countdown and related analyses report that heat-related deaths among adults aged over 65 in 2010s have already increased by more than 85% since the 1990s, and project substantial further increases in heat-related mortality in older age groups.^28^ A Projection study from China indicates that climate change will substantially increase the proportion of deaths attributable to non-optimal temperatures among older adults, and advanced aging is likely to increase mortality and offset the decline in cold-related mortality, with people above 80 years of age expected to suffer more from cold-related mortality.^25^

On this basis, we suggest that research and policy would benefit from more nuanced age stratification within older populations. Researchers could routinely report outcomes separately for at least young-old and old–old adults, exploring interactions between age, comorbidity, functional status and social determinants. Policymakers and practitioners in the WPR might consider ensuring that climate adaptation strategies are designed and monitored with explicit attention to the needs of old–old adults, so that this rapidly growing population is not only visible in high-level strategies but meaningfully protected as climate risks intensify.

## Limitations of this study

This review has several limitations related to its search strategy and to the generalizability of its evidence base. Regarding search strategy, the keyword-based approach, together with the absence of backward and forward citation searching, may have led to the omission of eligible studies. For example, those involving older adults without an exclusive focus on this population. Although the breadth of search terms across three major databases likely limited this risk. Relatedly, although air pollution and vector-borne/zoonotic disease search terms were required to co-occur with climate-related terms to ensure topical relevance, a precise conceptual boundary between climate-related and general epidemiological evidence remains difficult to establish given their interrelated nature; this requirement may also have excluded relevant studies not framed in explicitly climate-related language. Regarding generalizability, the evidence base was concentrated in a small number of settings, primarily mainland China, and restricted to English-language peer-reviewed literature; both factors limit how far these findings can be generalized across the Western Pacific Region.

Despite these limitations, this review provides a valuable foundation, identifying research gaps and informing future inquiries on climate change and older adults’ health in this region.

## Future research directions

This review reveals significant gaps in geographical coverage and threats related to climate change. The existing literature is predominantly concentrated in China and narrowly focused on extreme heat and cold. Future research should pursue more geographically balanced investigations across the WPR, with greater attention to long-term or cumulative climate exposures and their differential impacts across age subgroups among older adults. It would also be valuable to examine the conditions under which vulnerability of old–old adults is most pronounced, including interactions between climate stressors, health system factors, and social determinants, and to identify targeted adaptive strategies for this highest-risk group.

## Contributors

Dongkyu Lee: Writing—original draft, Writing—review & editing, Methodology, Investigation, Formal analysis, Data curation, Visualization, Funding acquisition.

In-Hwan Oh: Writing—original draft, Writing—review & editing, Formal analysis, Validation.

John S Ji: Writing—review & editing, Conceptualization, Validation.

Mikiko Kanda: Writing—review & editing, Validation.

Hitomi Kimura: Writing—review & editing, Validation.

Siwon Lee: Conceptualization, Writing—review & editing, Validation.

Jinah Park: Writing—review & editing, Investigation, Validation.

Josselyn Mothe: Writing—review & editing, Validation.

Enkhtsetseg Shinee: Writing—review & editing, Validation.

Ho Kim: Writing—review & editing, Project administration, Conceptualization, Supervision.

## Declaration of interests

Siwon Lee, Mikiko Kanda, Hitomi Kimura, Josselyn Mothe, and Enkhtsetseg Shinee are staff members of the World Health Organization (WHO). John S Ji is currently working at WHO as a visiting researcher from Tsinghua University. The authors alone are responsible for the views expressed in this article, which do not necessarily represent the decisions, policy, or views of WHO. The remaining authors declare no competing interests.
