# Supplementary material for: Age-stratified health impacts of climate change on older adults in the Western Pacific Region: a scoping review
Source: Lancet Reg Health West Pac. 2026 Oct 23;75:102004. doi: 10.1016/j.lanwpc.2026.102004 (PMC13721103; doi:10.1016/j.lanwpc.2026.102004)
Supplement: Supplementary file 1 [file mmc1.docx]

**Content**

**Table A1: Search terms used in the main review for English-language databases** ........................................ 1

**Table A2: Decision Rules for Age Gradient Classification by Tier**.................................................................. 4

**Table A3: Individual study-level effect estimates for climate-related threats and health outcomes, stratified by age, among older adults**................................................................................................................... 5

**Appendix A1: References included in this scoping review**.............................................................................. 18

**Table A1: Search terms used in the main review for English-language databases**

|  | **Medline (Pubmed)** |
| --- | --- |
| **1. Climate change** | ( "climate change"[tiab] OR "global warming"[tiab] OR "climate change"[MeSH Terms]  OR "sea-level rise"[tiab] OR "rising sea level"[tiab] OR "heat wave*"[tiab] OR "extreme heat"[tiab] OR "heat stress"[tiab] OR "cold wave*"[tiab] OR "cold spell*"[tiab] OR "temperature extreme*"[tiab] OR "Hot Temperature"[MeSH Terms] OR "Cold Temperature"[MeSH Terms] OR "Heat Stress Disorders"[MeSH Terms] OR "flood*"[tiab] OR "typhoon*"[tiab] OR "cyclone*"[tiab] OR "hurricane*"[tiab] OR "storm surge"[tiab] OR "Floods"[MeSH Terms] OR "wildfire*"[tiab] OR "bushfire*"[tiab] OR "forest fire*"[tiab] OR "Wildfires"[MeSH Terms] OR "Fires"[MeSH Terms] OR "drought*"[tiab] OR "Droughts"[MeSH Terms])  OR  (( "Air Pollution"[tiab] OR "Particulate Matter"[tiab] OR "PM2.5"[tiab] OR "PM 2.5"[tiab] OR "PM10"[tiab] OR "Ozone"[tiab] OR "Air Pollution"[MeSH Terms] OR "Particulate Matter"[MeSH Terms] OR "Ozone"[MeSH Terms] OR "vector borne disease*"[tiab] OR "zoonos*"[tiab] OR "Dengue"[tiab] OR "Malaria"[tiab] OR "Vector Borne Diseases"[MeSH Terms] OR "Zoonoses"[MeSH Terms] OR "Dengue"[MeSH Terms] OR "Malaria"[MeSH Terms]) AND ("climate change"[tiab] OR "climate variability"[tiab] OR "climate"[tiab] OR "climatic"[tiab] OR "global warming"[tiab] OR "climate change"[MeSH Terms])) |
| **2. Older adults** | "older adults"[tiab] OR "elderly"[tiab] OR "ageing"[tiab] OR "aging"[tiab] OR "senior"[tiab] OR "older people"[tiab] OR "aged"[MeSH Major Topic] |
| **3. The Western Pacific regions** | "American Samoa"[tiab] OR "Australia"[tiab] OR "Brunei"[tiab] OR "Cambodia"[tiab] OR "China"[tiab] OR "Cook Islands"[tiab] OR "Fiji"[tiab] OR "French Polynesia"[tiab] OR "Guam"[tiab] OR "Hong Kong"[tiab] OR "Hongkong"[tiab] OR "Indonesia"[tiab] OR "Japan"[tiab] OR "Kiribati"[tiab] OR "Lao"[tiab] OR "Laos"[tiab] OR "Macao"[tiab] OR "Malaysia"[tiab] OR "Marshall Islands"[tiab] OR "Micronesia"[tiab] OR "Mongolia"[tiab] OR "Nauru"[tiab] OR "New Caledonia"[tiab] OR "New Zealand"[tiab] OR "Niue"[tiab] OR "Northern Mariana Islands"[tiab] OR "Palau"[tiab] OR "Papua New Guinea"[tiab] OR "Philippines"[tiab] OR "Pitcairn Islands"[tiab] OR "Korea"[tiab] OR "Samoa"[tiab] OR "Singapore"[tiab] OR "Solomon Islands"[tiab] OR "Tokelau"[tiab] OR "Tonga"[tiab] OR "Tuvalu"[tiab] OR "Vanuatu"[tiab] OR "Viet Nam"[tiab] OR "Vietnam"[tiab] OR "Wallis and Futuna"[tiab] OR "Asia Pacific"[tiab] OR "Melanesia"[tiab] OR "Oceania"[tiab] OR "Pacific Islands"[tiab] OR "Polynesia"[tiab] OR "South Pacific"[tiab] OR "Western Pacific"[tiab] |
| **4. Period & Language** | 2016/01/01:2025/12/31[Date - Publication] AND "english"[Language] |
| **Scopus** | |
| **1. Climate change** | TITLE-ABS-KEY(  ("climate change" OR "global warming" OR "sea level rise" OR "sea-level rise" OR "rising sea level"  OR "heat wave*" OR "extreme heat" OR "heat stress" OR "cold wave*" OR "cold spell*" OR "temperature extreme*" OR "flood*" OR "typhoon*" OR "cyclone*" OR "hurricane*" OR "storm surge" OR "wildfire*" OR "bushfire*" OR "forest fire*" OR "drought*")  OR  (("air pollution" OR "particulate matter" OR "PM2.5" OR "PM 2.5" OR "PM10" OR "ozone"  OR "vector-borne disease*" OR "vectorborne disease*" OR "vector borne disease*" OR "zoonos*" OR "dengue" OR "malaria" ) AND ( "climate change" OR "climate variability" OR "climat*" OR "global warming" ))  ) |
| **2. Older adults** | TITLE-ABS-KEY ("older adults" OR "elderly" OR "ageing" OR "aging" OR "senior" OR "older people") |
| **3. The Western Pacific regions** | TITLE-ABS-KEY ( "American Samoa" OR "Australia" OR "Brunei" OR "Cambodia" OR "China" OR "Cook Islands" OR "Fiji" OR "French Polynesia" OR "Guam" OR "Hong Kong" OR "Hongkong" OR "Indonesia" OR "Japan" OR "Kiribati" OR "Lao" OR "Laos" OR "Macao" OR "Malaysia" OR "Marshall Islands" OR "Micronesia" OR "Mongolia" OR "Nauru" OR "New Caledonia" OR "New Zealand" OR "Niue" OR "Northern Mariana Islands" OR "Palau" OR "Papua New Guinea" OR "Philippines" OR "Pitcairn Islands" OR "Korea" OR "Samoa" OR "Singapore" OR "Solomon Islands" OR "Tokelau" OR "Tonga" OR "Tuvalu" OR "Vanuatu" OR "Viet Nam" OR "Vietnam" OR "Wallis and Futuna" OR "Asia Pacific" OR "Melanesia" OR "Oceania" OR "Pacific Islands" OR "Polynesia" OR "South Pacific" OR "Western Pacific" ) |
| **4. Period & Language** | PUBYEAR > 2015 AND PUBYEAR < 2026 |
| **Embase** | |
| **1. Climate change** | ( 'climate change'/exp OR 'climate change':ti,ab OR 'global warming':ti,ab OR 'sea level'/exp OR 'sea level rise':ti,ab OR 'sea-level rise':ti,ab OR 'rising sea level':ti,ab OR 'heat wave'/exp OR 'heat wave*':ti,ab OR 'extreme heat':ti,ab OR 'heat stress':ti,ab OR 'cold wave*':ti,ab OR 'cold spell*':ti,ab OR 'temperature extreme*':ti,ab OR 'flood'/exp OR 'flood*':ti,ab OR 'tropical cyclone'/exp OR 'typhoon*':ti,ab OR 'cyclone*':ti,ab OR 'hurricane*':ti,ab OR 'storm surge*':ti,ab OR 'wildfire'/exp OR 'wildfire*':ti,ab OR 'bushfire*':ti,ab OR 'forest fire*':ti,ab OR 'drought'/exp OR 'drought*':ti,ab)  OR  (( 'air pollution'/exp OR 'particulate matter'/exp OR 'ozone'/exp OR 'air pollution':ti,ab OR 'particulate matter':ti,ab OR 'pm2.5':ti,ab OR 'pm 2.5':ti,ab OR 'pm10':ti,ab OR 'ozone':ti,ab OR 'zoonosis'/exp OR 'dengue'/exp OR 'malaria'/exp OR 'vector borne disease'/exp OR 'vector-borne disease*':ti,ab OR 'vectorborne disease*':ti,ab OR 'vector borne disease*':ti,ab OR 'zoonos*':ti,ab OR 'dengue':ti,ab OR 'malaria':ti,ab) AND ( 'climate change'/exp OR 'climate change':ti,ab OR 'climate variability':ti,ab OR 'climate':ti,ab OR 'climatic':ti,ab OR 'global warming':ti,ab)) |
| **2. Older adults** | ('aged'/exp/mj OR 'older adults':ti,ab OR elderly:ti,ab OR ageing:ti,ab OR aging:ti,ab OR senior:ti,ab OR 'older people':ti,ab) |
| **3. The Western Pacific regions** | ('american samoa':ti,ab OR australia:ti,ab OR brunei:ti,ab OR cambodia:ti,ab OR china:ti,ab OR 'cook islands':ti,ab OR fiji:ti,ab OR 'french polynesia':ti,ab OR guam:ti,ab OR 'hong kong':ti,ab OR hongkong:ti,ab OR indonesia:ti,ab OR japan:ti,ab OR kiribati:ti,ab OR lao:ti,ab OR laos:ti,ab OR macao:ti,ab OR malaysia:ti,ab OR 'marshall islands':ti,ab OR micronesia:ti,ab OR mongolia:ti,ab OR nauru:ti,ab OR 'new caledonia':ti,ab OR 'new zealand':ti,ab OR niue:ti,ab OR 'northern mariana islands':ti,ab OR palau:ti,ab OR 'papua new guinea':ti,ab OR philippines:ti,ab OR 'pitcairn islands':ti,ab OR korea:ti,ab OR samoa:ti,ab OR singapore:ti,ab OR 'solomon islands':ti,ab OR tokelau:ti,ab OR tonga:ti,ab OR tuvalu:ti,ab OR vanuatu:ti,ab OR 'vietnam':ti,ab OR 'viet nam':ti,ab OR 'wallis and futuna':ti,ab OR 'asia pacific':ti,ab OR melanesia:ti,ab OR oceania:ti,ab OR 'pacific islands':ti,ab OR polynesia:ti,ab OR 'south pacific':ti,ab OR 'western pacific':ti,ab) |
| **4. Period & Language** | [2016-2025]/py AND [english]/lim |

**Table A2: Decision Rules for Age Gradient Classification by Tier**

| ***Tier*** | ***Basis for judgment*** | ***Labeled "Yes" when*** |
| --- | --- | --- |
| 3 | Formal test among older-adult subgroups | Older subgroup shows significantly stronger association |
| 2 | Formal test vs. younger reference (generally <65) | Only older subgroups are significant, or both significant with larger point estimate in older |
| 1 (CI reported) | Significance of subgroup-specific estimates | Only older subgroups are significant, or both significant with larger point estimate in older |
| 1 (no CI) | Point estimates only | Older subgroup shows larger point estimate |

*Note: For studies reporting three or more age subgroups, "older subgroup" refers to the oldest reported subgroup. The comparison is judged as significant if point estimates increase monotonically with age and are statistically significant, or if the oldest subgroup's estimate is substantially larger than the others' and statistically significant.*

**Table A3: Individual study-level effect estimates for climate-related threats and health outcomes, stratified by age, among older adults**

| **Threat** | **Outcome** | **Tier** | **Measure** | **Age Groups** | **Effect Estimates (point est, 95% CI)** | **Age-Group  Comparison** | **Age-Gradient**  **(tier 1 / 2-3)** | **Ref** |
| --- | --- | --- | --- | --- | --- | --- | --- | --- |
| Heat | Mortality | Tier 1 | OR | 65-74 75+ | 1.08 (0.92, 1.27), NS 1.23 (1.08, 1.41), sig | N/A | Yes / N/A | a2 |
| Heat | Mortality | Tier 1 | pooled RR | 65-74 75+ | 1.25 (1.15, 1.37), sig 1.67 (1.54, 1.82), sig | N/A | Yes / N/A | a9 |
| Heat | Mortality | Tier 1 | RR | 65-74 75+ | ~1.02 (~0.98, ~1.06), NS ~1.05 (~1.02, ~1.08), sig | N/A | Yes / N/A | a14 |
| Heat | Mortality | Tier 1 | RR | 65-74 75+ | 1.08 (1.05, 1.11), sig 1.12 (1.07, 1.17), sig | N/A | Yes / N/A | a20 |
| Heat | Mortality | Tier 1 | ER | 65-74 75+ | 2.61% (-1.59, 6.98), NS 3.00% (1.82, 4.19), sig | N/A | Yes / N/A | a21 |
| Heat | Mortality | Tier 1 | HR | 65-79 80-89 90-99 100+ | 1.04 (1.03, 1.05), sig 1.03 (1.02, 1.03), sig 1.03 (1.02, 1.04), sig 1.07 (1.05, 1.08), sig | N/A | Yes / N/A | a30 |
| Heat | Mortality | Tier 3 | Attributable YLL | 65-74  -  75+  - | 18.1 (14, 22.2), sig  -  58.9 (51.8, 66.0), sig  - | P < 0.05 (65-74 vs 75+) P < 0.05 (vs 0-64),  P < 0.05 (65-74 vs 75+) P < 0.05 (vs 0-64) | Yes / Yes | a38 |
| Heat | Mortality | Tier 1 | Cumulative RR | 65-69 70-74 75-79 80-84 85-89 90-94 95-99 100+ | 1.05 (1.00, 1.11), NS 1.05 (1.00, 1.10), NS 1.10 (1.06, 1.15), sig 1.11 (1.06, 1.15), sig 1.20 (1.14, 1.25), sig 1.32 (1.24, 1.42), sig 1.25 (1.14, 1.38), sig 1.15 (0.92, 1.46), NS | N/A | Yes / N/A | a39 |
| Heat | Mortality | Tier 1 | EMR | 60-74 (T_mean) 75-89 (T_mean) 90+ (T_mean) | 3.06% (1.86, 4.28), sig 3.78% (2.43, 5.14), sig 4.33% (2.28, 6.42), sig | N/A | Yes / N/A | a44 |
| Heat | Mortality | Tier 1 | RR | 65-74 75+ | 1.27 (1.09, 1.48), sig 1.71 (1.46, 1.99), sig | N/A | Yes / N/A | a50 |
| Heat | Mortality | Tier 1 | RR | 65-74 75-84 85+ | ~1.00 (~0.98, ~1.04), NS ~1.15 (~1.04, ~1.27), sig ~1.33 (~1.18, ~1.51), sig | N/A | Yes / N/A | a54 |
| Heat | Mortality | Tier 1 | RR | 60-64 65-79 80+ | 1.02 (0.98, 1.06), NS 1.06 (1.03, 1.08), sig 1.06 (1.04, 1.08), sig | N/A | Yes / N/A | a55 |
| Heat | Mortality | Tier 2 | OR | 65-74 75+ | 2.46 (2.02, 2.99), NS 5.06 (4.44, 5.76), sig | P=0.312 (ref: <65) P < 0.001 | Yes / Yes | a59 |
| Heat | Mortality | Tier 1 | ER | 65- (dry-hot) 85- (dry-hot) 65- (wet-hot) 85- (wet-hot) | 10.31% (8.86, 11.77), sig 17.96% (15.35, 20.62), sig 2.76% (-0.69, 6.32), NS 6.77% (1.11, 12.75), sig | N/A | Yes / N/A | a67 |
| Heat | Mortality | Tier 3 | % change | 65-74  -  75+  - | 26.1% (19.0, 33.6), sig  -  41.1% (30.5, 52.5), sig  - | P = 0.549 (0-64 vs 65-74) P = 0.023 (65-74 vs 75+) P = 0.011 (0-64 vs 65-74) P = 0.023 (65-74 vs 75+) | Yes / Yes | a68 |
| Heat | Mortality | Tier 1 | ASR | 50-69 70+ | 0.76 (no CI) 9.09 (no CI) | N/A | Yes / N/A | a70 |
| Heat | Mortality | Tier 2 | RR | 65-74 75+ | 1.22 (1.18, 1.26), sig 1.37 (1.32, 1.43), sig | P=0.024 (ref: <65) P < 0.001 | Yes / Yes | a74 |
| Heat | Mortality | Tier 1 | OR | 65-74 (night) 75+ (night) 65-74 (day) 75+ (day) | 1.18 (0.86, 1.63), NS 1.53 (1.34, 1.73), sig 1.61 (1.13, 2.28), sig 1.66 (1.44, 1.91), sig | N/A | Yes / N/A | a75 |
| Heat | Mortality | Tier 1 | pooled RR | 65-74 (`06-`19 pooled) 75+ (`06-`19 pooled) | 1.05 (0.87, 1.27), NS 1.21 (1.10, 1.33), sig | N/A | Yes / N/A | a82 |
| Heat | Mortality | Tier 1 | ER | 65-74 75-84 85-94 95+ | 0.49% (0.26, 0.71), sig 1.49% (1.19, 1.80), sig 1.79% (1.51, 2.07), sig 2.20% (1.51, 2.88), sig | N/A | Yes / N/A | a85 |
| Heat | Mortality | Tier 2 | RR | 65-74 75+ | 1.03 (0.94, 1.13), NS 1.11 (1.06, 1.17), sig | NS (omnibus test) sig | Yes / Yes | a87 |
| Heat | Mortality | Tier 3 | mortality risk | 75-85 85-95 >85 | 0.00023, NS 0.00105, sig 0.00216, sig | NS (ref: 65-74) P < 0.1 (ref: 65-74) P < 0.01 (ref: 65-74) | Yes / Yes | a91 |
| Heat | Mortality | Tier 1 | RR | 65-74 75-84 85+ | ~1.50 (~0.95, ~2.35), NS 1.76 (1.10, 2.81), sig 2.04: (1.37, 3.04), sig | N/A | Yes / N/A | a94 |
| Heat | Mortality | Tier 1 | temperature at RR 1.05 (°C; percentile) | 65-74 (Min) 75-84 (Min) 85+ (Min) | 29.1 °C (97.9 pct), NS 28.8 °C (95.4 pct), NS 28.5 °C (92.5 pct), sig | N/A | Yes / N/A | a96 |
| Heat | Mortality | Tier 2 | REOI | 75-85 85+ | 0.249 (0.139, 0.360), sig 0.315 (0.189, 0.441), sig | P < 0.05 (ref: < 75) P < 0.01 | Yes / Yes | a97 |
| Heat | Mortality | Tier 1 | CER | 65-84 (indoor) 85+ (indoor) 65-84 (outdoor) 85+ (outdoor) | 3.3% (2.8, 3.8), sig 8.3% (8.0, 8.7), sig 2.2% (2.0, 2.4), sig 4.7% (4.1, 5.2), sig | N/A | Yes / N/A | a99 |
| Heat | Mortality | Tier 1 | RR | 0-64 65-74 ≥75 | 1.12 (1.07, 1.17), sig 1.18 (1.12, 1.25), sig 1.31 (1.25, 1.37), sig | N/A | Yes / N/A | a34 |
| Heat | Mortality | Tier 1 | AF | 0-64 65-74 75+ | 0.5 (-0.2, 1.0), NS 1.3 (0.9, 1.6), sig 1.6 (1.3, 2.0), sig | N/A | Yes / N/A | a3 |
| Heat | Mortality | Tier 1 | AF | 65-74 75+ | -1.25% (-7.4, 5.01), NS 1.53% (-2.96, 5.09), NS | N/A | No / N/A | a18 |
| Heat | Mortality | Tier 2 | OR | 66-75 76-85 86-95 96+ | 1.129 (1.111, 1.146), NS 1.119 (1.091, 1.147), NS 1.147 (1.118, 1.176), NS 1.182 (1.085, 1.288), NS | P=0.321 (ref: <65) P=0.504 P=0.153 P=0.149 | No / No | a41 |
| Heat | Mortality | Tier 1 | OR | 65-74 (total accidental death) 75+ (total accidental death) | ~1.50 (~1.32, ~1.68), sig ~1.30 (~1.13, ~1.48), sig | N/A | No / N/A | a52 |
| Heat | Mortality | Tier 1 | CER | 50-69 70+ | 0.56 (0.31, 0.82), sig 0.23 (-0.18, 0.64), NS | N/A | No / N/A | a64 |
| Heat | Mortality | Tier 3 | COR | 60-74 75+ | 1.419 (1.305, 1.544), sig 1.411 (1.361, 1.463), sig | NS (60-74 vs 75+) NS (60-74 vs 75+) | No / No | a73 |
| Heat | Mortality | Tier 1 | COR | 60-74 75+ | 1.07 (1.01, 1.15), sig 1.05 (1.02, 1.07), sig | N/A | No / N/A | a76 |
| Heat | Mortality | Tier 3 | HR | 65-79 80-89 90-99 100+ | 1.82 (1.42,2.34), sig 1.75 (1.45,2.11), sig 1.82 (1.55,2.13), sig 1.80 (1.49,2.18), sig | From a separate model: HR for continuous age × heatwave interaction, 1.01 (0.99, 1.03), NS (P = 0.308; no ref) | No / No | a77 |
| Heat | Mortality | Tier 1 | RR | 65-74 (low O3) 75+ (low O3) 65-74 (high O3) 75+ (high O3) | 1.05 (1.01, 1.08), sig 1.03 (1.00, 1.05), sig 1.07 (1.02, 1.13), sig 1.06 (1.03, 1.09), sig | N/A | No / N/A | a80 |
| Heat | Mortality | Tier 3 | HR | 60-69 70-79 80+ | 1.06 (1.02, 1.11), sig 1.05 (1.01, 1.08), sig 1.03 (0.98, 1.08), NS | P = 0.725 (omnibus test) | No / No | a92 |
| Heat | Mortality | Tier 1 | Standardized β | 65-69 70-74 75-79 80-84 85+ | 0.290 (no CI), NS (P=.113) 0.335 (no CI), NS (P=.066) 0.410 (no CI), sig (P=.022) 0.014 (no CI), NS (P=.942) 0.335 (no CI), NS (P=.065) | N/A | No / N/A | a78 |
| Heat | Mortality | Tier 1 | RR | 65-74 (Nanjing) 75+ (Nanjing) 65-74 (Suzhou) 75+ (Suzhou) 65-74 (Yancheng 75+ (Yancheng) | ~1.20 (~1.02, ~1.42), sig 1.24 (1.16, 1.32), sig ~1.50 (~1.25, ~1.78), sig ~1.29 (1.21, 1.36), sig ~1.22 (~1.03, ~1.42), sig 1.42 (1.28, 1.58), sig | N/A | Mixed / N/A | a60 |
| Heat | Mortality | Tier 3 | OR | 65-74 (urban) 75+ (urban) 65-74 (rural) 75+ (rural) | 1.05 (0.99, 1.11), NS 1.11 (1.07,1.14), sig 1.13 (1.08, 1.17), sig 1.11 (1.06, 1.15), sig | P = 0.28 (ref: 75+, urban)  -  P = 1.00 (ref: 75+, rural)  - | Mixed / No | a72 |
| Heat | Mortality | Tier 2 | ER | 65-74 75+ | 13.27% (7.52, 19.32), sig 23.28% (18.23, 28.55), sig | P=0.22 (ref: < 64) p < 0.001 | Yes / Yes | a84 |
| Heat | Other health status | Tier 3 | HR | ~65-80  80+ | 1.047 (1.018, 1.076), sig 1.047 (1.033, 1.061), sig | P > 0.05 (interaction test) | No / No | a66 |
| Heat | Hospital admission | Tier 1 | RR | 60-74 75+ | 1.02 (0.89, 1.17), NS 1.20 (1.09, 1.31), sig | N/A | Yes / N/A | a10 |
| Heat | Hospital admission | Tier 2 | % increase per 1C | 60-74 75+ | 3.9% (3.4, 4.4), sig 4.3% (3.8, 4.8), sig | P=0.04 (ref: 0-59) P=0.01 | Yes / Yes | a40 |
| Heat | Hospital admission | Tier 1 | RR | 65-84 85+ | ~1.03 (~1.00, ~1.06), sig  ~1.04 (~1.01, ~1.07), sig | N/A | Yes / N/A | a51 |
| Heat | Hospital admission | Tier 1 | RR | 65-84 85+ | 1.07 (0.87, 1.33), NS 0.95 (0.59, 1.46), NS | N/A | No / N/A | a4 |
| Heat | Hospital admission | Tier 1 | pooled RR | 60-69 70-79 80+ | 1.01 (1.00, 1.03), sig 1.01 (1.00, 1.02), sig 1.00 (0.98, 1.01), NS | N/A | No / N/A | a28 |
| Heat | Hospital admission | Tier 1 | RR | 65-74 (OAH, lag 18) 75-84 (OAH, lag 13) 85+ (OAH, lag 16) | 1.28 (1.05-1.54), sig 1.11 (1.00-1.23), sig ~1.05 (~0.90, ~1.20), NS | N/A | No / N/A | a93 |
| Heat | Hospital admission | Tier 1 | % change | 60-69 (lag 0-3) 70-79 (lag 0-3) 80+ (lag 0-3) | +31.6% (no CI), sig -12.7% (no CI), NS +53.9% (no CI), sig | N/A | No / N/A | a69 |
| Heat | Emergency service | Tier 1 | RR | 60-79 (85th) 80+ (85th) 60-79 (95th) 80+ (95th) | ~1.04 (~0.99, ~1.10), NS ~1.08 (~1.01, ~1.15), sig ~1.07 (~1.01, ~1.13), sig ~1.11 (~1.03, ~1.19), sig | N/A | Yes / N/A | a12 |
| Heat | Emergency service | Tier 1 | RR | 65-74 (Low SVI) 75+ (Low SVI) 65-74 (Moderate SVI) 75+ (Moderate SVI) 65-74 (High SVI) 75+ (High SVI) | 1.017 (0.998, 1.036), NS 1.022 (1.009, 1.036), sig 1.016 (0.998, 1.034), NS 1.036 (1.018, 1.053), sig 1.024 (1.011, 1.037), sig 1.037 (1.028, 1.047), sig | N/A | Yes / N/A | a81 |
| Heat | Emergency service | Tier 1 | OR | 65-84 (MA) 85+ (MA) 65-84 (non-MA) 85+ (non-MA) | 1.09 (1.02, 1.16), sig 1.12 (1.01, 1.26), sig 1.00 (0.92, 1.09), NS 1.07 (0.92, 1.25), NS | N/A | Yes / N/A | a89 |
| Heat | Emergency service | Tier 1 | OR | 65-74 75-84 85+ | 1.070 (1.027, 1.116), sig 1.106 (1.067, 1.147), sig 1.117 (1.061, 1.177), sig | N/A | Yes / N/A | a100 |
| Heat | Emergency service | Tier 3 | % change | 60-74 75+ | -  0.43 (-0.95, 1.83), NS | P = 0.698 (ref: 60-74) | No / No | a24 |
| Heat | Emergency service | Tier 1 | RR | 60-74 (Warm days) 75+ (Warm days) | 1.04 (1.01, 1.08), sig 1.02 (0.98, 1.06), NS | N/A | No / N/A | a27 |
| Heat | Emergency service | Tier 1 | IRR | ≥60 years (T_max) ≥65 years (T_max) ≥70 years (T_max) | 1.224 (sig, p<0.001) 1.204 (sig, p<0.001) 1.178 (sig, p<0.001) | N/A | No / N/A | a31 |
| Heat | Emergency service | Tier 1 | RR | 60-74 (examination) 75+ (examination) | ~1.05 (~0.85, ~1.30), NS ~0.83 (~0.60, ~1.15), NS | N/A | No / N/A | a33 |
| Heat | Emergency service | Tier 1 | OR | 65-74 (max temp≥37C) 75+ (max temp≥37C) | 15.47 (6.60, 36.48), sig 12.90 (7.06, 23.57), sig | N/A | No / N/A | a37 |
| Heat | Emergency service | Tier 1 | RR | 50-69 (at 30°C) 70+ (at 30°C) | ~1.43 ~1.02 | N/A | No / N/A | a47 |
| Heat | Other service use | Tier 3 | OR | 60-69 70-79 80+ | 1.06 (0.98, 1.15), NS 1.10 (0.99, 1.23), NS 1.38 (1.22, 1.56), sig | NS (ref: 2-sample test, not specified) NS (ref: " ) P < 0.05 (ref: " ) | Yes / Yes | a98 |
| Cold | Mortality | Tier 1 | CER | 65-74 75-84 85+ | ~23 (~12, ~33), sig ~20 (~8, ~32), sig ~50 (~33, ~68), sig | N/A | Yes / N/A | a5 |
| Cold | Mortality | Tier 2 | OR | 65-74 75-84 85+ | 1.04 (0.95, 1.15), NS 1.19 (1.10, 1.30), sig 1.33 (1.22, 1.45), sig | NS (ref: <65) sig sig | Yes / Yes | a6 |
| Cold | Mortality | Tier 1 | AF | 65-74 75+ | 3.52% (-3.45, 9.37), NS 5.34% (0.44, 9.38), sig | N/A | Yes / N/A | a18 |
| Cold | Mortality | Tier 2 | RR | 65-74 75+ | 1.46 (1.33, 1.59), sig 1.62 (1.42, 1.81), sig | (Z-test not applied)  P=0.015 (ref: 0-64) | Yes / Yes | a19 |
| Cold | Mortality | Tier 1 | ER | 65-74 75+ | 1.75% (0.82, 2.69), sig 3.04% (2.47, 3.61), sig | N/A | Yes / N/A | a21 |
| Cold | Mortality | Tier 3 | Attributable YLL | 65-74  - 75+  - | 1.5 (0.7, 2.2), sig  -  5.5 (4.2, 6.8), sig  - | P < 0.05 (65-74 vs 75+) P < 0.05 (vs 0-64),  P < 0.05 (65-74 vs 75+) P < 0.05 (vs 0-64) | Yes / Yes | a38 |
| Cold | Mortality | Tier 1 | Cumulative RR | 65-69 70-74 75-79 80-84 85-89 90-94 95-99 100+ | 1.06 (0.99, 1.16), NS 1.12 (1.06, 1.21), sig 1.11 (1.06, 1.17), sig 1.20 (1.14, 1.26), sig 1.23 (1.16, 1.30), sig 1.36 (1.25, 1.48), sig 1.34 (1.18, 1.52), sig 1.47 (1.15, 1.92), sig | N/A | Yes / N/A | a39 |
| Cold | Mortality | Tier 1 | EMR | 60-74 (T_mean) 75-89 (T_mean) 90+ (T_mean) | 1.34% (1.07, 1.62), sig 2.19% (1.91, 2.47), sig 3.63% (3.07, 4.20), sig | N/A | Yes / N/A | a44 |
| Cold | Mortality | Tier 1 | RR | 65-74 75-84 85+ | ~1.25 (~1.01, ~1.55), sig ~1.34 (~1.16, ~1.55), sig ~1.41 (~1.21, ~1.64), sig | N/A N/A N/A | Yes / N/A | a54 |
| Cold | Mortality | Tier 2 | RR | 65-74 (dry cold) 75-84 (dry cold) 85+ (dry cold) 65-74 (wet-cold) 75-84 (wet-cold 85+ (wet-cold) | 1.08 (1.03, 1.14), sig 1.11 (1.06, 1.17), sig 1.16 (1.10, 1.22), sig 1.21 (1.15, 1.28), sig 1.25 (1.20, 1.31), sig 1.34 (1.27, 1.41), sig | P = 0.89 (ref: 0-64, dry cold) P = 0.18 (ref: 0-64, dry cold) P = 0.005 (ref: 0-64, dry cold) P = 0.08 (ref: 0-64, wet-cold) P = 0.001 (ref: 0-64, wet-cold) P < 0.001 (ref: 0-64, wet-cold) | Yes / Yes | a65 |
| Cold | Mortality | Tier 1 | CRR | 60-74 75-89 89+ | ~1.32 (1.13, 1.53), sig ~1.50 (1.30, 1.72), sig ~1.57 (1.31, 1.87), sig | N/A | Yes / N/A | a71 |
| Cold | Mortality | Tier 3 | COR | 60-74 75+ | 1.07 (0.99, 1.15), NS 1.18 (1.14, 1.22), sig | sig (60-74 vs 75+) | Yes / Yes | a76 |
| Cold | Mortality | Tier 1 | pooled RR | 65-74 (`06-`19 pooled) 75+ (`06-`19 pooled) | 1.15 (1.02, 1.30), sig 1.27 (1.13, 1.42), sig | N/A | Yes / N/A | a82 |
| Cold | Mortality | Tier 1 | ER | 65-74 75-84 85-94 95+ | 0.23% (-0.54, 1.00), NS 0.36% (0.16, 0.56), sig 0.54% (0.34, 0.74), sig 1.05% (0.51, 1.59), sig | N/A | Yes / N/A | a85 |
| Cold | Mortality | Tier 1 | RR | 65-74 75+ | 1.46 (1.37, 1.56), sig 1.81 (1.72, 1.91), sig | N/A | Yes / N/A | a95 |
| Cold | Mortality | Tier 1 | CER | 65-84 (indoor) 85+ (indoor) 65-84 (outdoor) 85+ (outdoor) | 1.2% (1.0, 1.4), sig 1.8% (1.6, 2.1), sig 0.9% (0.8, 0.9), sig 1.0% (0.9, 1.1), sig | N/A | Yes / N/A | a99 |
| Cold | Mortality | Tier 1 | OR | 65-74 (Lag6, P10) 75-84 (Lag6, P10) ≥85 (Lag6, P10) | ~1.06 (~0.98, ~1.15), NS ~1.08 (~1.00, ~1.14), NS ~1.14 (~1.06, ~1.22), sig | N/A | Yes / N/A | a43 |
| Cold | Mortality | Tier 1 | OR | 65-74 75-84 85+ | 1.11 (0.66, 1.89), NS 0.96 (0.57, 1.59), NS 1.11 (0.74, 1.67), NS | N/A | No / N/A | a25 |
| Cold | Mortality | Tier 1 | CRR | 65-74 75-84 85+ | 1.102 (0.928, 1.307), NS 1.205 (1.055, 1.377), sig 1.081 (0.964, 1.213), NS | N/A | No / N/A | a79 |
| Cold | Mortality | Tier 1 | RR | 65-74 (low O3) 75+ (low O3) 65-74 (high O3) 75+ (high O3) | 1.06 (1.03, 1.09), sig 1.04 (1.02, 1.06), sig 1.04 (0.99, 1.10), NS 1.03 (1.00, 1.05), sig | N/A | No / N/A | a80 |
| Cold | Mortality | Tier 1 | AF | 65-74 75+ | 14.9 (10.2, 18.2), sig 14.0 (10.4, 16.7), sig | N/A | No / N/A | a3 |
| Cold | Hospital admission | Tier 1 | AF | 65-74 (CBI) 75+ (CBI) 65-74 (ICH) 75+ (ICH) | 9.6% (-0.9, 16.6), NS 12.7% (5.0, 19.2), sig 13.0% (-11.9, 25.0), NS 26.1% (14.4, 32.4), sig | N/A | Yes / N/A | a8 |
| Cold | Hospital admission | Tier 1 | pooled RR | 60-69 70-79 80+ | 0.98 (0.97, 1.00), NS 1.03 (1.01, 1.04), sig 1.07 (1.06, 1.09), sig | N/A | Yes / N/A | a28 |
| Cold | Hospital admission | Tier 1 | RR | 65-84 (total CVD) 85+ (total CVD) | ~1.50 (~1.40, ~1.61), sig ~1.66 (~1.52, ~1.82), sig | N/A | Yes / N/A | a51 |
| Cold | Emergency service | Tier 1 | AOR | 65-74 75+ | 1.46 (0.62, 3.45), NS 3.07 (1.55, 6.09), sig | N/A | Yes / N/A | a11 |
| Cold | Emergency service | Tier 1 | RR | 60-74 (Cold nights) 75+ (Cold nights) | 1.02 (1.00, 1.04), sig 1.04 (1.02, 1.08), sig | N/A | Yes / N/A | a27 |
| Cold | Emergency service | Tier 1 | RR | 60-74 (examination) 75+ (examination) | ~1.15 (~0.85, ~1.55), NS 1.41 (1.05, 1.89), sig | N/A | Yes / N/A | a33 |
| Cold | Emergency service | Tier 1 | OR | 65-84 (MA) 85+ (MA) 65-84 (non-MA) 85+ (non-MA) | 1.39 (1.27, 1.53), sig 1.40 (1.19, 1.64), sig 1.35 (1.19, 1.53), sig 1.49 (1.19, 1.86), sig | N/A | Yes / N/A | a89 |
| Cold | Emergency service | Tier 1 | OR | 65+ (vs <65 ref) 85+ (vs <85 ref) | 3.69 (1.2, 11), sig 3.37 (1.5, 7.9), sig | N/A | No / N/A | a23 |
| Cold | Emergency service | Tier 1 | RR | 50-69 (-10°C) 70+ (-10°C) | ~1.25 ~1.22 | N/A | No / N/A | a47 |
| Cold | Other service use | Tier 3 | OR | 60-69 70-79 80+ | 1.07 (1.01, 1.14), NS 1.25 (1.16, 1.35), sig 1.60 (1.48, 1.73), sig | NS (ref: 2-sample test, not specified) P < 0.05 (ref: " ) P < 0.05 (ref: " ) | Yes / Yes | a98 |
| TV | Mortality | Tier 3 | AF | 65-74 75+ | 2.48% (0.33, 5.30), sig  -  5.78% (3.53, 8.04), sig  - | P = 0.002 (65-74 vs 75+) P < 0.233 (vs 0-64),  P = 0.002 (65-74 vs 75+) P < 0.001 (vs 0-64) | Yes / Yes | a53 |
| TV | Mortality | Tier 1 | LLD | 65-74 75+ | 0.85 (0.40, 1.31), sig 0.40 (0.21, 0.59), sig | N/A | No / N/A | a61 |
| TV | Mortality | Tier 1 | RR | 60-74 75-89 90+ | 2.492 (1.757, 3.535), sig 1.947 (1.449, 2.617), sig 1.560 (0.987, 2.466), NS | N/A | No / N/A | a86 |
| TV | Other health status | Tier 3 | OR | 60-65 65-70 70+ | 1.06 (0.94, 1.20), NS 1.17 (1.01, 1.36), sig 1.06 (0.92, 1.23), NS | P = 0.535 (omnibus test) | No / No | a88 |
| TV | Other health status | Tier 1 | OR | 60-74 75+ | ~1.18 (~1.13, ~1.22), sig ~1.14 (~1.07, ~1.21), sig | N/A | No / N/A | a63 |
| TV | Hospital admission | Tier 2 | % change | 65-74 75+ | 0.55 (0.34, 0.75), sig 0.81 (0.59, 1.03), sig | P=0.008 (ref: 18-64) P=0.001 | Yes / Yes | a17 |
| TV | Hospital admission | Tier 2 | % change | 65-74 75+ | 0.73% (0.30, 1.16), NS 0.76% (0.37, 1.15), NS | P=0.493 (ref: 18-64) P=0.412 | No / No | a48 |
| TV | Emergency service | Tier 3 | % change | 65-74 (Male) 75+ (Male) 65-74 (Female) 75+ (Female) | 1.33 (-0.24, 2.92), NS 1.31 (0.11, 2.52), sig 0.74 (-0.29, 1.78), NS 2.23 (1.24, 3.22), sig | NS (Male, 65-74 vs 75+) NS (Male, 65-74 vs 75+) sig p < 0.05 (Female, 65-74 vs 75+) sig p < 0.05 (Female, 65-74 vs 75+) | Yes / Mixed | a1 |
| TV | Emergency service | Tier 2 | ER | 65-74 75+ | 1.80% (1.17, 2.43), sig 1.80% (1.03, 2.58), sig | Z-test, sig (ref: <65) Z-test, sig | No / No | a36 |
| Extreme Weather | Mortality | Tier 1 | Fatality rate | 60-69 (Male) 70-79 (Male) 80+ (Male) 60-69 (Female) 70-79 (Female) 80+ (Female) | ~0.0025 (no CI) ~0.0014 (no CI)  ~0.0119 (no CI) ~0.0022 (no CI)  ~0.0030 (no CI)  ~0.0061 (no CI) | N/A | Yes / N/A | a49 |
| Heat | Mortality | Tier 2 | ER | 65-74 75+ | 13.27% (7.52, 19.32), sig 23.28% (18.23, 28.55), sig | P=0.22 (ref: < 64) p < 0.001 | Yes / Yes | a84 |
| Extreme Weather | Mortality | Tier 1 | RR | 65-74 (lag 13) 75-84 (lag 13) ≥85 (lag 13) | ~1.20 (1.06, 1.42), sig ~1.10 (~0.90, ~1.35), NS ~1.15 (~0.85, ~1.50), NS | N/A | No / N/A | a83 |
| Extreme Weather | Other health status | Tier 3 | OR | 65-75 75+ (ref) | 1.32 (0.86, 2.02), NS - | P = 0.20 (ref: 75+) | No / No | a62 |
| Extreme Weather | Other health status | Tier 3 | Adjusted HR | 65-79 80+ | 1.22 (0.84, 1.78), NS 1.24 (1.05, 1.48), sig | P = 0.98 (omnibus test) | Yes / No | a58 |
| Extreme Weather | Other health status | Tier 3 | Bushfire impact score (median, IQR) | 65-69 70-74 75+ | 2.8 (no CI) 2.0 (no CI) 1.7 (no CI) | P = 0.003 (65–69 vs 75+)  - (not reported) P = 0.003 (65–69 vs 75+)  P = 0.003 (omnibus test) | No / No | a57 |
| Extreme Weather | Other service use | Tier 3 | Adjusted HR | ~65-80 80+ | 2.55 (2.20, 2.96), sig 1.91 (1.75, 2.10), sig | P = 0.0002 (ref: ~65-80) | No / No | a42 |
| Extreme Weather | Other service use | Tier 3 | HR | 65-69 70-74 75-79 80-84 85-89 90-94 94+ | 5.40 (2.62, 11.10), sig 4.87 (2.89, 8.21), sig 3.77 (2.56, 5.55), sig 3.21 (2.40, 4.30), sig 3.43 (2.80, 4.20), sig 3.08 (2.42, 3.91), sig 2.30 (1.57, 3.39), sig | P = 0.05 (omnibus test) | No / No | a56 |
| Pollution | Mortality | Tier 1 | % of EM | 60-64 (East Asia) 65-69 (East Asia) 70-74 (East Asia) 75-79 (East Asia) 80+ (East Asia) 60-64 (Australia) 65-69 (Australia) 70-74 (Australia) 75-79 (Australia) 80+ (Australia) | ~8 (no CI) ~9 (no CI) ~10 (no CI) ~13 (no CI) ~17 (no CI) ~8 (no CI) ~9 (no CI) ~10 (no CI) ~13 (no CI) ~23 (no CI) | N/A | Yes / N/A | a26 |
| Pollution | Mortality | Tier 1 | % increase | 65-74 (PM_25) 74+ (PM_25) 65-74 (EC) 74+ (EC) | 2.24 (−0.27, 4.81), NS 0.59 (−0.76, 1.96), NS 2.68 (0.44, 4.96), sig 0.53 (-0.67, 1.74), NS | N/A | No / N/A | a13 |
| Pollution | Mortality | Tier 1 | HR | 65-74 75-84 85-94 95+ | 1.93 (1.777, 2.096), sig 1.554 (1.477, 1.635), sig 1.243 (1.2, 1.287), sig 1.062 (1.026, 1.099), sig | N/A | No / N/A | a46 |
| Pollution | Other health status | Tier 1 | OR | 60-69 70-84 85+ | ~1.75 (1.40–2.15), sig ~1.85 (1.50–2.25), sig ~1.95 (1.35–2.80), sig | N/A | Yes / N/A | a90 |
| Pollution | Other health status | Tier 3 | Adjusted HR | 65-79 80+ | 1.160 (1.031, 1.305), sig 1.086 (1.020, 1.157), sig | P = 0.300 (ref: 65-79) | No / No | a45 |
| Pollution | Hospital admission | Tier 1 | % change | 51-74 75+ | ~0.8% (~-0.4, ~1.9), NS ~1.6% (~0.3, ~2.9), sig | N/A | Yes / N/A | a32 |
| Pollution | Hospital admission | Tier 1 | % increase | 66-85 >85 | 0.30 (0.11, 0.50), sig -0.31 (-0.60, -0.02), sig | N/A | No / N/A | a7 |
| Pollution | Hospital admission | Tier 1 | % increase | 60-70 (PM2.5) 70-80 (PM2.5) 80+ (PM2.5) 60-70 (PM10) 70-80 (PM10) 80+ (PM10) | ~3.4 (~2.0, ~4.8), sig ~2.7 (~1.6, ~3.7), sig 3.01 (1.97, 4.06), sig ~1.8 (~1.0, ~2.7), sig ~1.65 (~1.0, ~2.3), sig 1.82 (1.17, 2.46), sig | N/A | No / N/A | a15 |
| Pollution | Hospital admission | Tier 2 | % increase | 65-74 (PM2.5) 75+ (PM2.5) 65-74 (PM10) 75+ (PM10) | 0.35 (0.12, 0.58), NS 0.42 (0.23, 0.62), NS 0.20 (0.10, 0.30), NS 0.32 (0.22, 0.39), sig | P=0.479 (ref: 18-64) P=0.201 P=0.126 P < 0.001 | Mixed / Yes | a16 |
| Pollution | Emergency service | Tier 1 | RR | 60-74 75+ | ~1.0016 (~1.0001, ~1.0032), sig ~1.0017 (~1.0002, ~1.0035), sig | N/A | Yes / N/A | a29 |
| Pollution | Other service use | Tier 3 | ER | 65-74 (RESD) 75+ (RESD) 65-74 (CCD) 75+ (CCD) | 0.40 (-0.14, 0.95), NS -0.11 (-0.68, 0.47), NS 0.53 (0.02, 1.04), sig 0.87 (0.37, 1.39), sig | NS (vs 75+), NS (vs 5-64) NS (vs 65-74), NS (vs 5-64) NS (vs 75+), NS (vs 5-64) NS (vs 65-74), NS (vs 5-64) | Mixed / No | a22 |

*Notes. AF, attributable fraction (%); ASR, age-standardized rate; CER, cumulative excess risk; COR: cumulative odds ratio, CRR, cumulative relative risk; EM, excess mortality (%); ENP, expected number per population; ER, excess risk (ER = RR − 1); HR, hazard ratio; LLD, life loss per death; OR, odds ratio; PC, percent (%); RR, relative risk; YLL, years of life lost; Asterisks (*) in the “Effect size” column denotes statistical significance of each estimate denoted in original paper. ~ means value visually estimated from figure (paper reported results graphically only, no exact numerical estimates given), Age Gradient: Whether vulnerability (risk/effect) increases with advancing age within the older-adult population studied.*

**Appendix A2. References included in this scoping review**

a1.	Zheng S, Wang M, Li B, et al. Gender, Age and Season as Modifiers of the Effects of Diurnal Temperature Range on Emergency Room Admissions for Cause-Specific Cardiovascular Disease among the Elderly in Beijing. International Journal of Environmental Research and Public Health 2016; 13(5): 447.

a2.	Zhang J, Liu S, Han J, et al. Impact of heat waves on nonaccidental deaths in Jinan, China, and associated risk factors. International Journal of Biometeorology 2016; 60(9): 1367–75.

a3.	Yang J, Yin P, Zhou M, et al. The burden of stroke mortality attributable to cold and hot ambient temperatures: Epidemiological evidence from China. Environment International 2016; 92-93: 232–8.

a4.	Phung D, Guo Y, Thai P, et al. The effects of high temperature on cardiovascular admissions in the most populous tropical city in Vietnam. Environmental Pollution 2016; 208: 33–9.

a5.	Wang L, Liu T, Hu M, et al. The impact of cold spells on mortality and effect modification by cold spell characteristics. Scientific Reports 2016; 6: 38380.

a6.	Qiu H, Tian L, Ho K-f, Yu IT, Thach T-Q, Wong C-M. Who is more vulnerable to death from extremely cold temperatures? A case-only approach in Hong Kong with a temperate climate. International Journal of Biometeorology 2016; 60(5): 711–7.

a7.	Xu A, Mu Z, Jiang B, et al. Acute effects of particulate air pollution on ischemic heart disease hospitalizations in Shanghai, China. International Journal of Environmental Research and Public Health 2017; 14(2): 168.

a8.	Guo P, Zheng M, Feng W, et al. Effects of ambient temperature on stroke hospital admissions: results from a time-series analysis of 104,432 strokes in Guangzhou, China. Science of the Total Environment 2017; 580: 307–15.

a9.	Zhou L, Chen K, Chen X, et al. Heat and mortality for ischemic and hemorrhagic stroke in 12 cities of Jiangsu Province, China. Science of the Total Environment 2017; 601: 271–7.

a10.	Chan EYY, Lam HCY, So SHW, et al. Association between Ambient Temperatures and Mental Disorder Hospitalizations in a Subtropical City: A Time-Series Study of Hong Kong Special Administrative Region. International Journal of Environmental Research and Public Health 2018; 15(4).

a11.	Matsuyama T, Morita S, Ehara N, et al. Characteristics and outcomes of accidental hypothermia in Japan: the J-Point registry. Emergency Medicine Journal 2018; 35(11): 659–66.

a12. Kotani K, Ueda K, Seposo X, et al. Effects of high ambient temperature on ambulance dispatches in different age groups in Fukuoka, Japan. Global Health Action 2018; 11(1): 1437882.

a13.	Chen C, Xu D, He MZ, et al. Fine particle constituents and mortality: a time-series study in Beijing, China. Environmental Science & Technology 2018; 52(19): 11378.

a14.	Yin P, Chen R, Wang L, et al. The added effects of heatwaves on cause-specific mortality: a nationwide analysis in 272 Chinese cities. Environment International 2018; 121: 898–905.

a15.	Qiu H, Tan K, Long F, et al. The burden of COPD morbidity attributable to the interaction between ambient air pollution and temperature in Chengdu, China. International Journal of Environmental Research and Public Health 2018; 15(3): 492.

a16.	Tian Y, Liu H, Wu Y, et al. Ambient particulate matter pollution and adult hospital admissions for pneumonia in urban China: a national time series analysis for 2014 through 2017. PLoS Medicine 2019; 16(12): e1003010.

a17.	Tian Y, Liu H, Si Y, et al. Association between temperature variability and daily hospital admissions for cause-specific cardiovascular disease in urban China: a national time-series study. PLoS Medicine 2019; 16(1): e1002738.

a18.	Deng C, Ding Z, Li L, et al. Burden of non-accidental mortality attributable to ambient temperatures: a time series study in a high plateau area of southwest China. BMJ Open 2019; 9(2): e024708.

a19.	Chen J, Yang J, Zhou M, et al. Cold spell and mortality in 31 Chinese capital cities: definitions, vulnerability and implications. Environment International 2019; 128: 271–8.

a20.	Yang J, Yin P, Sun J, et al. Heatwave and mortality in 31 major Chinese cities: definition, vulnerability and implications. Science of The Total Environment 2019; 649: 695–702.

a21.	Lin Q, Lin H, Liu T, et al. The effects of excess degree-hours on mortality in Guangzhou, China. Environmental Research 2019; 176: 108510.

a22.	Wang C, Feng L, Chen K. The impact of ambient particulate matter on hospital outpatient visits for respiratory and circulatory system disease in an urban Chinese population. Science of The Total Environment 2019; 666: 672–9.

a23.	Forcey DS, FitzGerald MP, Burggraf MK, Nagalingam V, Ananda-Rajah MR. 'Cold and lonely': emergency presentations of patients with hypothermia to a large Australian health network. Internal Medicine Journal 2020; 50(1): 54–60.

a24.	Wang Y, Liu Y, Ye D, et al. High temperatures and emergency department visits in 18 sites with different climatic characteristics in China: Risk assessment and attributable fraction identification. Environment International 2020; 136: 105486.

a25.	Ho HC, Chan T-C, Xu Z, Huang C, Li C. Individual- and community-level shifts in mortality patterns during the January 2016 East Asia cold wave associated with a super El Niño event: Empirical evidence in Hong Kong. Science of the Total Environment 2020; 711: 135050.

a26.	Lelieveld J, Pozzer A, Pöschl U, Fnais M, Haines A, Münzel T. Loss of life expectancy from air pollution compared to other risk factors: a worldwide perspective. Cardiovascular Research 2020; 116(11): 1910–7.

a27.	Ma Y, Jiao H, Zhang Y, et al. Short-term effect of extreme air temperature on hospital emergency room visits for cardiovascular diseases from 2009 to 2012 in Beijing, China. Environmental Science and Pollution Research 2020; 27(30): 38029–37.

a28.	Lu P, Xia G, Zhao Q, Xu R, Li S, Guo Y. Temporal trends of the association between ambient temperature and hospitalisations for cardiovascular diseases in Queensland, Australia from 1995 to 2016: a time-stratified case-crossover study. PLoS Medicine 2020; 17(7): e1003176.

a29.	Zhang Y, Ma Y, Feng F, et al. Association between PM10 and specific circulatory system diseases in China. Scientific Reports 2021; 11(1): 12129.

a30.	Zhang H, Liu L, Zeng Y, Liu M, Bi J, Ji JS. Effect of heatwaves and greenness on mortality among Chinese older adults. Environmental Pollution 2021; 290: 118009.

a31. Toosty NT, Hagishima A, Tanaka K-I. Heat health risk assessment analysing heatstroke patients in Fukuoka City, Japan. PLoS One 2021; 16(6): e0253011.

a32.	Hu W, Chen Y, Chen J. Short-term effect of fine particular matter on daily hospitalizations for ischemic stroke: A time-series study in Yancheng, China. Ecotoxicology and Environmental Safety 2021; 208: 111518.

a33.	Wang Y, Liu Y, Ye D, et al. Temperatures and health costs of emergency department visits: a multisite time series study in China. Environmental Research 2021; 197: 111023.

a34.	He G, Xu Y, Hou Z, et al. The assessment of current mortality burden and future mortality risk attributable to compound hot extremes in China. Science of The Total Environment 2021; 777: 146219.

a35.	Li Z, Hu J, Meng R, et al. The association of compound hot extreme with mortality risk and vulnerability assessment at fine-spatial scale. Environmental Research 2021; 198: 111213.

a36.	Zhu W, Wei X, Zhang L, et al. The effect and prediction of diurnal temperature range in high altitude area on outpatient and emergency room admissions for cardiovascular diseases. International Archives of Occupational and Environmental Health 2021; 94(8): 1783–95.

a37.	Otani S, Funaki Ishizu S, Masumoto T, Amano H, Kurozawa Y. The effect of minimum and maximum air temperatures in the summer on heat stroke in Japan: a time-stratified case-crossover study. International Journal of Environmental Research and Public Health 2021; 18(4): 1632.

a38.	Zeng W, Yu M, Mai W, et al. Age-specific disparity in life loss per death attributable to ambient temperature: A nationwide time-series study in China. Environmental Research 2022; 203: 111834.

a39.	Huang Y, Yang J, Chen J, Shi H, Lu X. Association between ambient temperature and age-specific mortality from the elderly: Epidemiological evidence from the Chinese prefecture with most serious aging. Environmental Research 2022; 211: 113103.

a40.	Lu P, Xia G, Zhao Q, et al. Attributable risks of hospitalizations for urologic diseases due to heat exposure in Queensland, Australia, 1995–2016. International Journal of Epidemiology 2022; 51(1): 144–54.

a41.	Pan R, Zheng H, Ding Z, et al. Attributing hypertensive life expectancy loss to ambient heat exposure: a multicenter study in eastern China. Environmental Research 2022; 208: 112726.

a42. Miyamori D, Yoshida S, Kashima S, Koike S, Ishii S, Matsumoto M. Discontinuation of long-term care among persons affected by the 2018 Japan Floods: A longitudinal study using the Long-term Care Insurance Comprehensive Database. BMC Geriatrics 2022; 22(1): 168.

a43.	Du J, Cui L, Ma Y, et al. Extreme cold weather and circulatory diseases of older adults: A time-stratified case-crossover study in Jinan, China. Environmental Research 2022; 214: 114073.

a44.	Bi X, Wu C, Wang C, et al. Impacts of air temperature and its extremes on human mortality in Shanghai, China. Urban Climate 2022; 41: 101072.

a45.	Gao Q, Zang E, Bi J, et al. Long-term ozone exposure and cognitive impairment among Chinese older adults: A cohort study. Environment International 2022; 160: 107072.

a46.	Ji JS, Liu L, Zhang J, et al. NO2 and PM2.5 air pollution co-exposure and temperature effect modification on pre-mature mortality in advanced age: a longitudinal cohort study in China. Environmental Health 2022; 21(1): 97.

a47.	Dawson LP, Andrew E, Nehme Z, et al. Temperature-related chest pain presentations and future projections with climate change. Science of The Total Environment 2022; 848: 157716.

a48.	Tian Y, Wu J, Liu H, et al. Ambient temperature variability and hospital admissions for pneumonia: A nationwide study. Science of The Total Environment 2023; 856: 159294.

a49.	Fan J, Huang G. Are Women More Vulnerable to Flooding Than Men in an Aging Japanese Society? International Journal of Environmental Research and Public Health 2023; 20(2): 1299.

a50.	Zhou Y, Gao Y, Yin P, et al. Assessing the Burden of Suicide Death Associated With Nonoptimum Temperature in a Changing Climate. JAMA Psychiatry 2023; 80(5): 488–97.

a51.	Pan R, Okada A, Yamana H, et al. Association between ambient temperature and cause-specific cardiovascular disease admissions in Japan: A nationwide study. Environmental Research 2023; 225: 115610.

a52.	He C, Yin P, Chen R, et al. Cause-specific accidental deaths and burdens related to ambient heat in a warming climate: A nationwide study of China. Environment International 2023; 180: 108231.

a53.	Liu J, Du X, Yin P, Kan H, Zhou M, Chen R. Cause-specific mortality and burden attributable to temperature variability in China. Science of The Total Environment 2023; 896: 165267.

a54.	Xia Y, Shi C, Li Y, et al. Effects of ambient temperature on mortality among elderly residents of Chengdu city in Southwest China, 2016–2020: a distributed-lag non-linear time series analysis. BMC Public Health 2023; 23(1): 149.

a55.	Franklin RC, Mason HM, King JC, et al. Heatwaves and mortality in Queensland 2010–2019: implications for a homogenous state-wide approach. International Journal of Biometeorology 2023; 67(3): 503–15.

a56.	Miyamori D, Yoshida S, Kashima S, et al. How the 2018 Japan Floods Impacted Nursing Home Admissions for Older Persons: A Longitudinal Study Using the Long-Term Care Insurance Comprehensive Database. Journal of the American Medical Directors Association 2023; 24(3): 368–75.e1.

a57.	Halcomb E, Thompson C, Morris D, et al. Impacts of the 2019/20 bushfires and COVID-19 pandemic on the physical and mental health of older Australians: a cross-sectional survey. Family Practice 2023; 40(3): 449–57.

a58.	Ikeda K, Yoshida S, Okazaki Y, et al. Increased care-need in older long-term care insurance users after the 2018 Japan Floods: a retrospective cohort study based on the Japanese long-term care insurance claims. Environmental Health and Preventive Medicine 2023; 28: 31.

a59.	Cao Y, Lu Z, Chu J, et al. Intraseasonal variation of the association between heat exposure and mortality risk in Shandong province, China. Urban Climate 2023; 51: 101621.

a60.	Wang L. Mediating Effect of Heat Waves between Ecosystem Services and Heat-Related Mortality of Characteristic Populations: Evidence from Jiangsu Province, China. International Journal of Environmental Research and Public Health 2023; 20(3): 2750.

a61.	Gong W, Li X, Zhou M, et al. Mortality burden attributable to temperature variability in China. Journal of Exposure Science & Environmental Epidemiology 2023; 33(1): 118–24.

a62.	Patrick R, Snell T, Gunasiri H, Garad R, Meadows G, Enticott J. Prevalence and determinants of mental health related to climate change in Australia. Australian & New Zealand Journal of Psychiatry 2023; 57(5): 710–24.

a63.	Wen B, Su BB, Xue J, et al. Temperature variability and common diseases of the elderly in China: a national cross-sectional study. Environmental Health 2023; 22(1): 4.

a64.	Hu J, He G, Meng R, et al. Temperature-related mortality in China from specific injury. Nature Communications 2023; 14(1): 37.

a65. Li M, Fang W, Meng R, et al. The comparison of mortality burden between exposure to dry-cold events and wet-cold events: A nationwide study in China. Science of The Total Environment 2023; 904: 166859.

a66. Zhou W, Wang Q, Li R, et al. The effects of heatwave on cognitive impairment among older adults: Exploring the combined effects of air pollution and green space. Science of The Total Environment 2023; 904: 166534.

a67.	Fang W, Li Z, Gao J, et al. The joint and interaction effect of high temperature and humidity on mortality in China. Environment International 2023; 171: 107669.

a68.	Niu Y, Yang J, Zhao Q, et al. The main and added effects of heat on mortality in 33 Chinese cities from 2007 to 2013. Frontiers of Environmental Science & Engineering 2023; 17(7): 81.

a69.	Yong KH, Teo YN, Azadbakht M, Phung H, Chu C. The Scorching Truth: Investigating the Impact of Heatwaves on Selangor's Elderly Hospitalisations. International Journal of Environmental Research and Public Health 2023; 20(10): 5910.

a70.	Zheng J, Lin H, Ling J, Huang J, Li D. The trends of disease burden due to high temperature in Mainland China from 1990 to 2019 and its prediction to 2030. Scientific Reports 2023; 13(1): 22238.

a71.	Song H-J, Cheng Y-B, Wang Y, et al. Total and cause-specific mortality attributable to cold spells in China: A multicity and multicounty study. Advances in Climate Change Research 2023; 14(6): 827–35.

a72.	Tao J, Zheng H, Ho HC, et al. Urban-rural disparity in heatwave effects on diabetes mortality in eastern China: A case-crossover analysis in 2016–2019. Science of The Total Environment 2023; 858: 160026.

a73.	Zhang R, Sun L, Jia A, et al. Effect of heatwaves on mortality of Alzheimer's disease and other dementias among elderly aged 60 years and above in China, 2013–2020: a population-based study. The Lancet Regional Health – Western Pacific 2024; 52.

a74.	Du H, Yan M, Liu X, et al. Exposure to Concurrent Heatwaves and Ozone Pollution and Associations with Mortality Risk: A Nationwide Study in China. Environmental Health Perspectives 2024; 132(4): 47012.

a75.	Gao Y, Lin L, Yin P, Kan H, Chen R, Zhou M. Heat Exposure and Dementia-Related Mortality in China. JAMA Network Open 2024; 7(6): e2419250.

a76.	Zhang R, Sun L, Jia A-N, et al. Impact of ambient temperatures on Alzheimer's disease and other dementia mortality among elderly patients aged 60 years and older in China. Advances in Climate Change Research 2024; 15(6): 1088–95.

a77.	Xi D, Liu L, Zhang M, et al. Risk factors associated with heatwave mortality in Chinese adults over 65 years. Nature Medicine 2024; 30(5): 1489–98.

a78.	Park C-Y. The 2018 Heat Wave's Impact on the Mortality of Older People in Seoul, South Korea. Social Work in Public Health 2024; 39(5): 478–96.

a79.	Jin S, Bi X, Wu C, et al. The associations of cold spells with human mortality in Shanghai, China. Urban Climate 2024; 58: 102175.

a80.	Tajudin MABA, Kubo R, Ng CFS, et al. The effect modification of PM2.5 and ozone on the short-term associations between temperature and mortality across the urban areas of Japan. Environmental Health and Preventive Medicine 2024; 29: 57.

a81.	Ho JY-e, Lai ET, Chau PH, Chong KC, Woo J. The role of older adult-focused social vulnerability on the relationship between temperature and emergency department attendance in a subtropical Asian city. Archives of Gerontology and Geriatrics 2024; 117: 105195.

a82.	Qi J, Zhang J, Wang Y, et al. The temporal variation in the effects of extreme temperature on respiratory mortality: Evidence from 136 cities in China, 2006–2019. Environment International 2024; 189: 108800.

a83.	Ma Y, Zhang X, Zhang Y, et al. The threaten of typhoons to the health of residents in inland areas: a study on the vulnerability of residents to death risk during typhoon "Lekima". BMC Public Health 2024; 24(1): 606.

a84.	He G, Lin Y, Hu J, et al. The trends of non-accidental mortality burden attributed to compound hot-dry events in China and its provinces in a global warming world. Environment International 2024; 191: 108977.

a85.	Lin Y, Qi J, Hu J, et al. Age inequality in temperature-related fall mortality among old people in China in a warming climate. npj Climate and Atmospheric Science 2025; 8(1): 58.

a86.	Zha Q, Chai G, Zhang L, Cheng L, Li Y, Yao X. All-cause and cause-specific mortality attributable to diurnal temperature range in Ningbo City, China: a time-series study based on 188,039 death records, 2014–2018. Air Quality, Atmosphere & Health 2025; 18(12): 4093–107.

a87.	Wang Y, Lin C, Lau AKH, Fung JCH, Chong KC, Ren C. Assessing the impact of compound heatwave-ozone event with varied definitions on mortality risk in Hong Kong. Environment International 2025; 201: 109595.

a88.	Zhong Y, Wang S, Hao D, et al. Association between Diurnal Temperature Range, Daily Mean Temperature and Sarcopenia States in Older Adults: Evidence from the CHARLS. BMC Geriatrics 2025; 25(1): 531.

a89.	Kim A, Park J, Kang C, Kim H, Lee W. Double disparities of the excess risks and costs of extreme temperatures on hospitalization between Medical Aid and non-Medical Aid populations in South Korea. International Journal of Epidemiology 2025; 54(2).

a90.	Li Y, Shen X, Chen C, Zhong P, Su B, Zheng X. Effect modification of heatwaves on the association of long-term ozone exposure and cardiovascular disease among older adults in China: A national representative cross-sectional study. Journal of Hazardous Materials Advances 2025; 17: 100585.

a91.	Yao X, Qu Y, Mishra AK, et al. Elderly vulnerability to temperature-related mortality risks in China. Science Advances 2025; 11(6): eado5499.

a92.	Wu Y, Feng X, Li J, et al. Exposure to high-temperature and high-humidity environments associated with cardiovascular mortality. Ecotoxicology and Environmental Safety 2025; 290: 117746.

a93.	Lai ET-C, Ho IY-Y, Ho HC, et al. Extreme Hot Weather Events and Risk of Hospitalization for Cardiovascular and Respiratory Diseases in Older People in Hong Kong in 2012–2018. The Journals of Gerontology: Series A 2025; 80(4).

a94.	Zheng L, Wang J, Su M, Huang Q, Jiang C. Matrix–patch effects of vegetation and urban parks: Their modification role on heat-related mortality. Urban Forestry & Urban Greening 2025; 114: 129136.

a95.	Jiang Z, Ji X, Zhuo Y, et al. Mortality risk and burden attributable to compound cold extreme in China: A national time series study. Environment International 2025; 197: 109364.

a96.	Ho JY-e, Guo Y, Chong KC, et al. Suitable temperature indicator for adverse health impacts in sub-tropical cities: a case study in Hong Kong from 2010–2019. International Journal of Biometeorology 2025; 69(1): 233–44.

a97.	Wang Z, Zhu L, Peng M, Zheng H, Zhang Y. Summer heatwave, ozone pollution and ischemic stroke mortality: An individual-level case-crossover study. Environmental Research 2025; 268: 120818.

a98.	Ning Z, Ma Y, He S, et al. The interactive effect of extreme weather events and PM2.5 on respiratory health among the elderly: a case-crossover study in a high-altitude city. International Journal of Biometeorology 2025; 69(2): 331–42.

a99.	Huang Z, Hu J, Gao J, et al. The mortality burden attributed to regional indoor temperatures in China. Environment International 2025; 204: 109822.

a100.	Xu Z, Rutherford S, Nghiem S, et al. The odds and costs of ambulance attendances associated with heatwave severity in older adults of Queensland, Australia. International Journal of Biometeorology 2025; 69(10): 2661–8.
